# Structure–function analysis of the *Fusarium oxysporum* Avr2 effector allows uncoupling of its immune‐suppressing activity from recognition

**DOI:** 10.1111/nph.14733

**Published:** 2017-08-31

**Authors:** Xiaotang Di, Lingxue Cao, Richard K. Hughes, Nico Tintor, Mark J. Banfield, Frank L. W. Takken

**Affiliations:** ^1^ Molecular Plant Pathology SILS University of Amsterdam PO Box 94215 1090 GE Amsterdam the Netherlands; ^2^ Department of Biological Chemistry John Innes Centre Norwich Research Park Norwich NR4 7UH UK

**Keywords:** effector, Fusarium wilt, plant immunity, PRR‐triggered immunity, *Pseudomonas syringae*, *Verticillium dahlia*

## Abstract

- Plant pathogens employ effector proteins to manipulate their hosts. *Fusarium oxysporum* f. sp. *lycopersici* (*Fol*), the causal agent of tomato wilt disease, produces effector protein Avr2. Besides being a virulence factor, Avr2 triggers immunity in *I‐2* carrying tomato (*Solanum lycopersicum)*. *Fol* strains that evade *I‐2* recognition carry point mutations in *Avr2* (e.g. Avr2^R45H^), but retain full virulence.
- Here we investigate the virulence function of Avr2 and determine its crystal structure. Transgenic tomato and Arabidopsis expressing either wild‐type *ΔspAvr2* (deleted signal‐peptide) or the *ΔspAvr2* ^*R45H*^ variant become hypersusceptible to fungal, and even bacterial infections, suggesting that Avr2 targets a conserved defense mechanism. Indeed, *Avr2* transgenic plants are attenuated in immunity‐related readouts, including flg22‐induced growth inhibition, ROS production and callose deposition.
- The crystal structure of Avr2 reveals that the protein shares intriguing structural similarity to ToxA from the wheat pathogen *Pyrenophora tritici‐repentis* and to TRAF proteins. The I‐2 resistance‐breaking Avr2^V41M^, Avr2^R45H^ and Avr2^R46P^ variants cluster on a surface‐presented loop. Structure‐guided mutagenesis enabled uncoupling of virulence from I‐2‐mediated recognition.
- We conclude that I‐2‐mediated recognition is not based on monitoring Avr2 virulence activity, which includes suppression of immune responses via an evolutionarily conserved effector target, but by recognition of a distinct epitope.

## Introduction

The soil‐inhabiting fungus *Fusarium oxysporum* causes vascular wilt disease on a wide range of plants, provoking severe economic losses. Although *F. oxysporum* species have been reported to collectively infect > 120 different hosts, each *formae speciales* (f.sp.) is specific to a unique host (Michielse & Rep, 2009). Of all *F. oxysporum* pathosystems, the interaction between *F. oxysporum* f.sp. *lycopersici* (*Fol*) and tomato is among the best studied, and represents an excellent model system to study the molecular mechanisms underlying plant disease and resistance (Takken & Rep, 2010). During infection, *Fol* attaches to the root surfaces of its host, penetrates and colonizes the plant xylem vessels (di Pietro *et al*., 2003). Subsequent blockage of the vasculature prevents transport of water and nutrients causing the typical wilt symptoms after which this disease is named.

Plants have evolved a multilayered immune system to halt pathogens and prevent disease (Chisholm *et al*., 2006). At the cell surface, immune receptors (pattern recognition receptors (PRRs)) survey the environment for conserved signatures of pathogens, and when activated initiate a defense response (PRR‐triggered immunity (PTI); Jones & Dangl, 2006). Flagellin Sensing 2 (FLS2) receptor, one of the best‐characterized PRRs, mediates recognition of bacterial flagellin (or its derived elicitor‐active peptide flg22) (Gomez‐Gomez & Boller, 2000).

Like other PRRs, FLS2 forms complexes with other cell‐surface proteins (in this case the BRI1‐Associated Receptor Kinase 1 (BAK1)) in the presence of its ligand to initiate various immune responses (Boller & Felix, 2009). Early responses typically include the rapid and transient production of reactive oxygen species (ROS), activation of mitogen‐activated protein kinases (MAPK), transcriptional reprogramming and stomatal closure (Melotto *et al*., 2006). Late responses, such as callose deposition or seedling growth inhibition, develop within longer periods ranging from hours to days (Nicaise *et al*., 2009). Successful pathogens typically have to overcome such immune responses to establish a successful infection. One mechanism that host‐adapted pathogens use to achieve this is the production of virulence factors – called effectors – that can inhibit PRR complexes or their downstream signaling events. To halt such pathogens, plants have evolved a set of receptors that recognize effectors, or their actions, and induce immunity to prevent the spread of disease. As many effectors act inside host cells the majority of these receptors are localized intracellularly and mostly belong to the family of Nucleotide binding domain, Leucine rich repeat Receptors (NLRs) (Jones *et al*., 2016). To date, three of these receptor genes (also referred to as plant disease resistance genes), *I*,* I‐2* and *I‐3,* have been introgressed from wild tomato species into cultivated tomato (*Solanum lycopersicum*) to confer resistance against *Fol* races 1, 2 and 3, respectively. Whereas *I* and *I‐3* encode extracellular receptors, I‐2 is a classical intracellular NLR (Simons *et al*., 1998; Catanzariti *et al*., 2015, 2017).

Candidate *Fol* effector proteins identified in xylem sap of tomato plants have been named Six (Secreted in Xylem) proteins (Rep *et al*., 2004; Houterman *et al*., 2008). Avr3 (Avirulence factor 3, Six1) is a virulence factor that triggers *I‐3*‐mediated resistance. Avr1 (Six4) induces *I*‐mediated resistance and suppresses *I‐2*‐ and *I‐3*‐mediated disease resistance (Rep *et al*., 2005; Houterman *et al*., 2008). Avr2 is required for *Fol* virulence in susceptible tomato varieties, but triggers resistance in plants carrying *I‐2* (Houterman *et al*., 2009). *Fol* race 3 strains carry point mutations in Avr2 that do not affect its virulence activity, but allow it to evade *I‐2*‐mediated recognition (Houterman *et al*., 2009). The *Avr2* (*Six3*) gene encodes a 15.7 kDa protein (after processing of the signal peptide) that contains two cysteine residues (Houterman *et al*., 2007). The Avr2 protein is recognized by I‐2 inside the plant nucleus (Ma *et al*., 2013) and exerts its virulence function inside host cells (Di *et al*., 2016). Avr2 shares no sequence homology to known proteins (including effectors) (Houterman *et al*., 2009), and it has not been possible to predict Avr2 biochemical functions based on protein sequence.

In order to gain further insights into the molecular mechanisms underlying the virulence activity of Avr2, we constitutively expressed a cytosolic version of the wild‐type *Avr2* and the *Avr2* ^*R45H*^ variant in tomato and in Arabidopsis. Here, we report that heterologous expression of both the *ΔspAvr2* and *ΔspAvr2* ^*R45H*^ variant promoted susceptibility towards various bacterial and fungal plant pathogens in both hosts. Consistent with the potential for Avr2 to target a conserved defense mechanism in different hosts, we demonstrate that *Avr2* suppresses PRR (FLS2)‐mediated responses, such as growth inhibition, ROS production, MAPK activation and callose deposition following flagellin22 (flg22) treatment. In an attempt to resolve the structural requirements underlying I‐2‐mediated recognition of Avr2, and its relationship to virulence, we determined the crystal structure of this effector. Structure‐informed site‐directed mutagenesis of Avr2, combined with functional analyses of the variants, allowed us to identify residues that are critical for its virulence activity, and to uncouple virulence from I‐2‐mediated immune recognition.

## Materials and Methods

### Plant material and fungal and bacterial strains

Tomato (*Solanum lycopersicum*) cv Moneymaker was used. Tomato seeds were grown in soil with 16 h : 8 h, light : dark cycles, at 22 : 16°C, day : night and 70% relative humidity in a glasshouse. Arabidopsis seedlings were grown under short day conditions, 13 h : 11 h, dark : light cycles at 22°C. The pathogenic strains *Verticillium dahliae* race 1 JR2 (Fradin *et al*., 2009), *Botrytis cinerea* strain B05.10 (Zhang & Van Kan, 2013), *Fusarium oxysporum* (*Fo)5176* (Thatcher *et al*., 2012), and *Pseudomonas syringae* pv. *tomato* (*Pst*) DC3000 (Whalen *et al*., 1991) and *Fo* f.sp. *lycopersici* (*Fol*) ***Δ**Avr2* (Houterman *et al*., 2009) have been described previously.

### Vector construction

*ΔspAvr2* ^*R45H*^ was amplified with primers FP2525 and FP2274 using CTAPi::*ΔspAvr2* ^*R45H*^ as template (Houterman *et al*., 2009). The product was cloned into SLDB3104 (Tameling *et al*., 2010) in a similar way to SLDB3104::*ΔspAvr2* (Ma *et al*., 2013). Site‐directed *ΔspAvr2* mutants were generated using quick‐change mutagenesis (Zheng *et al*., 2004), using pDONR207::*ΔspAvr2* as template (Houterman *et al*., 2009). The products were introduced into the cTAPi vector (Rohila *et al*., 2004) by gateway cloning. All PCR primers were purchased from MWG (http://www.mwg-biotech.com) and listed in Supporting Information Table S1. All plasmids have been confirmed by sequence analysis.

### Plant transformation

Moneymaker was transformed with SLDB3104::*ΔspAvr2* ^*R45H*^ in a similar way to that described for SLDB3104::*ΔspAvr2* transformation (Di *et al*., 2016). Two homozygous single copy insertion lines from 17 independent T2 generations were selected according to segregation for kanamycin resistance. Presence of *Avr2* was confirmed by PCR (primers FP962 and FP963) and by Western blot. Arabidopsis Col‐0 was transformed by floral dipping (Clough & Bent, 1998). Transformants were selected on medium containing kanamycin, timentin and nystatin (40, 100 and 100 mg l^−1^, respectively) and transferred to soil. Nine independent single insertion lines were selected according to segregation analysis on selective plates, and three homozygous T3 lines were selected. Presence of the transgene was confirmed by PCR (primers FP872 and FP873).

### RNA isolation and reverse transcription polymerase chain reaction (RT‐PCR) to verify *Avr2* expression in Arabidopsis

Leaf material (± 400 mg rosette) from 14‐d‐old Arabidopsis seedlings was ground in liquid nitrogen. Total RNA was extracted using TRIzol LS reagent (Invitrogen) and DNA was removed by on‐column RNase‐free DNase (Qiagen) treatment. cDNA was synthesized from 1 μg of total RNA with a M‐MulV reverse‐transcriptase RNaseH minus kit with oligo dT primers (Fermentas). Arabidopsis actin (primers FP3147 and FP3148) was used as internal control for RT‐PCR. Primer pair FP2849/FP2848 was used to amplify *Avr2* from cDNA.

### Infection assays of Arabidopsis and tomato

*Fusarium oxysporum* and *V. dahliae* inoculation of Arabidopsis and tomato seedlings was done by dipping the roots in a fungal spore suspension (10^6^ spores ml^−1^). After repotting disease symptoms were recorded after 2–3 wk (Supporting Information Methods S1). For *B. cinerea* inoculation, droplets of a fungal spore suspension (5 × 10^6^ conidia ml^−1^) were placed on detached leaves of 5‐wk‐old plants. Lesion diameters at 3 d post inoculation (dpi) were quantified by ImageJ (Methods S1). *Pst* DC3000 inoculation was done by syringe‐infiltration of a bacterial suspension (OD_600_ of 0.0005) into leaves of 4‐wk‐old tomato or *Arabidopsis thaliana* plants. Bacterial titers were determined for 0–3 dpi (Methods S1).

### Flg22‐induced tomato seedlings growth inhibition assay

Flagellin22 (flg22)‐induced seedling growth inhibition assays (Gomez‐Gomez *et al*., 1999) were performed as described previously (Pfund *et al*., 2004). Tomato seeds were sterilized for 2 min in 3% hypochlorite and 10 min in 70% ethanol, and subsequently washed three times in sterile H_2_O. Seeds pre‐germinated for 48 h on 1% water agar and 10 seedlings were transferred to a 250‐ml glass flask containing 200 ml of ½ MS and 1% (w/v) sucrose liquid media supplemented with either 100 nM flg22 or water. The seedlings were incubated at 21**°**C with 12 h : 12 h, light : dark while shaking at 100 rpm. FW and root length were recorded after 7 d.

### Callose deposition

Callose was visualized as described (Gomez‐Gomez *et al*., 1999). Leaf discs taken from tomato seedlings treated with flg22 as described above were incubated with 70% ethanol for 1 h and subsequently with 100% ethanol until clear. Cleared leaves were rehydrated sequentially for 30 min in 50% ethanol. For *A. thaliana* 10‐d‐old seedlings were transferred to 0.5× Murashige & Skoog liquid medium supplemented with water or with 100 nM flg22 24 h before sampling. Arabidopsis seedlings and tomato leaf disks were stained with a 0.01% aniline blue solution in 150 mM K_2_HPO4 at pH 9.5 for 30–120 min and mounted in 50% or 70% glycerol (respectively) for callose visualization using an EVOS FL microscope (Thermo Fisher Scientific, Carlsbad, CA, USA) using DAPI channel (UV fluorescence). Callose foci within the frame of a single image (magnification ×4) were counted by ImageJ (https://imagej.net/Welcome).

### Oxidative burst assay

Reactive oxygen species (ROS) measurement was performed based on a luminol/peroxidase‐based assay (Felix *et al*., 1999). Leaf discs of 4‐wk‐old tomato, Arabidopsis or *Nicotiana benthamiana* (diameter = 5 mm) were collected with a cork borer and floated in sterile water overnight. The leaf discs were transferred to 96‐well plate containing 100 μl water supplied with 250 μM luminol and 10 μg ml^−1^ horseradish peroxidase (HRP). The luminescence was recorded over a 50‐min period using Magellan F50 (Tecan) after the treatment of 100 nM flg22 or water and then displayed as the sum of photon counts.

### Mitogen‐activated protein kinase (MAPK) assay

Leaf discs (diameter = 5 mm) from 4‐wk‐old *N. benthamiana* plants were floated on sterile water overnight and then exposed to 0.5 μM flg22 for 0, 5 and 15 min. Total protein was isolated from 20 mg plant material and separated on 10% SDS‐PAGE gels and blotted on polyvinylidene fluoride (PVDF) membranes as described (Flury *et al*., 2013). The primary antibody (a‐p44/42 MAPK, monoclonal D13.14.4E, Cell Signaling Technology (Bioké), Leiden, the Netherlands) was used at a 1 : 5000 dilution and the secondary antibody (goat‐anti‐rabbit, Pierce 31460) at a 1 : 3000 dilution. Membranes were probed with the ECL plus kit from Thermo Scientific.

### *Agrobacterium*‐mediated transient transformation of *N. benthamiana*

*Agrobacterium tumefaciens* strain GV3101 was transformed with binary constructs and used for transient transformation as described (Ma *et al*., 2012). Leaves of 4–5‐wk‐old plants were infiltrated with a suspension with an A_600_ of 0.2 (for *I‐2* constructs) or 0.5 (for *Avr2* constructs). For ROS and MAPK assays the silencing suppressor strain P19 was co‐infiltrated at A_600_ of 0.5.

### Trypan blue staining

Leaves were boiled for 5 min in a 1 : 1 mixture of 96% ethanol and staining solution (100 ml lactic acid, 100 ml phenol, 100 ml glycerol, 100 ml H_2_O and 100 mg Trypan blue). Leaves were destained in 2.5 g ml^−1^ chloral hydrate in water (Ma *et al*., 2012).

### Protein extraction and Western blotting

Protein extraction was carried out as described (Ma *et al*., 2015). Twenty microliter samples were run on 13% SDS–PAGE gels and blotted on PVDF membranes using semi‐dry blotting. The membranes were subjected to immunoblotting using either anti‐HA peroxidase at a dilution of 1 : 3000 (clone 3F10; Roche) or anti‐Avr2 antibody (1 : 5000 diluted) (Ma *et al*., 2015). The secondary antibody goat‐anti‐rat (P31470, Pierce) was used at a 1 : 5000 dilution. Luminescence was visualized by ECL using BioMax MR film.

### Heterologous gene expression in *E. coli*, and protein purification

*Avr2* lacking the N‐terminal 37 amino acids, corresponding to the protein identified in the xylem sap (Ma *et al*., 2013), was cloned into pOPINS3C (Berrow *et al*., 2007) by In‐Fusion cloning (Clontech, Takara Bio Europe SAS, Saint‐Germain‐en‐Laye, France). Avr2 was produced in *E. coli* SHuffle cells (NEB) (Bessette *et al*., 1999). Bacterial cultures were grown in Luria‐Bertani media to an *A*_600_ 0.5–0.8, then protein expression was induced with 1 mM isopropyl‐1‐thio‐β‐d‐galactopyranoside and incubated at 18°C for 18 h. Following centrifugation, cell pellets were resuspended in 50 mM Tris‐HCl buffer, pH 8.0 containing 0.5 M NaCl, 50 mM glycine, 5% (v/v) glycerol, 20 mM imidazole and protease inhibitors (Complete EDTA‐free tablets, 1 tablet per 50 ml, Roche). Cells were lysed by sonication. His_6_‐SUMO‐tagged Avr2 was purified from cleared lysate using an ÄKTAxpress system (GE Healthcare, Little Chalfont, UK) by IMAC (Immobilized Metal Affinity Chromatography) on a 5 ml Ni^2+^ His‐Trap FF column followed by gel filtration on a Superdex 75 26/60 column pre‐equilibrated in 20 mM HEPES, pH 7.5 containing 0.15 M NaCl. Fractions containing Avr2 protein, as judged by SDS‐PAGE, were incubated overnight at 4°C with 3C protease (10 μg mg^−1^ protein) to remove the His_6_‐SUMO‐tag. Avr2 protein was separated from uncleaved protein and His_6_‐SUMO‐tag by IMAC chromatography, then concentrated to 5–7 ml and repurified by gel filtration as above. Avr2 protein was concentrated to 16 mg mg^−1^ and frozen in 50 μl aliquots in liquid nitrogen and stored at −80°C. Avr2 protein concentration was estimated using predicted extinction coefficients for the His_6_‐SUMO fusion and cleaved proteins of 18 910 M^−1^ cm^−1^ and 17 420 M^−1^ cm^−1^, respectively.

### Crystallization, data collection and structure determination

Crystallization experiments were performed using an Oryx Nano robot (Douglas Instruments, Hungerford, UK). Crystals grew in 0.1 M MES, pH 6.5 with 25% (w/v) PEG 8K. Before X‐ray data collection, crystals were transferred into a cryoprotectant solution containing 0.1 M MES, pH 6.5 containing 30% (w/v) PEG 8K and 20% (v/v) ethylene glycol, mounted in a litho loop and flash‐frozen in liquid nitrogen. Some crystals were soaked for ~ 60 s in well solution supplemented with 0.5 M potassium iodide Before flash freezing as above. Native and SAD (single wavelength anomalous diffraction) X‐ray datasets were collected at the Diamond Light Source, UK, on beamline I03 (under proposal mx4975) (Table 1). The data were processed using the xia2 pipeline (Winter, 2010). The structure was solved using the SAD approach with the data collected from a crystal soaked in potassium iodide solution. Iodide sites were identified with Phenix (Adams *et al*., 2010). These positions were used to estimate initial phases using Crank2 (Skubak & Pannu, 2013) and Phaser (McCoy *et al*., 2007) from the CCP4 suite (Winn *et al*., 2011). An initial model was built using Buccaneer (Cowtan, 2006). The final model was produced through iterative rounds of refinement using Refmac5 (Murshudov *et al*., 1997) and manual rebuilding with Coot (Emsley *et al*., 2010). Structure validation used the tools provided in Coot and Molprobity (Chen *et al*., 2010).

**Table 1:** X‐ray data collection and refinement statistics

	AVR2	
	Native	Iodide
Data collection statistics		
Wavelength (Å)	0.92	1.90
Space group	*P*2_1_2_1_2_1_	*P*2_1_2_1_2_1_
Cell dimensions		
*a*,* b*,* c* (Å)	33.41, 51.73, 74.79	33.59, 51.50, 74.91
Resolution (Å)^a^	42.54–1.10 (1.12–1.10)	74.91–2.40 (2.46–2.40)
*R*_*merge*_ (%)	5.4 (28.5)	8.2 (15.9)
*I/*σ*I*	26.1 (6.3)	38.0 (20.8)
Completeness (%)		
Overall	99.8 (95.6)	100 (100)
Anomalous		100 (99.7)
Unique reflections	53 312 (2506)	5458 (378)
Redundancy		
Overall	12.8 (6.2)	25.6 (21.8)
Anomalous		13.9 (11.3)
*CC* (1/2) (%)	99.9 (96.1)	99.9 (99.4)
Refinement and model statistics		
Resolution (Å)	40.00–1.10 (1.13–1.10)	
*R*_work_/*R*_free_ (%)	13.9/16.9 (13.7/15.2)	
No. of atoms		
Protein/ligands/waters	1028/20/154	
*B*‐Factors (Å^2^)		
Protein/ligands/waters	11.8/21.9/26.8	
Root mean square deviations		
Bond lengths (Å)	0.014	
Bond angles (°)	1.82	
Ramachandran plot (%)^a^		
Favoured	99.2	
Allowed	0.8	
Outliers	0.0	
Molprobity score	0.96 (98^th^ percentile)	

The highest resolution shell is shown in parentheses.

a Data are as calculated by Molprobity.

## Results

### Expression of *Avr2* ^*R45H*^ in tomato plants confers hypersusceptibility to *V. dahliae*,* B. cinerea* and *Pseudomonas syringae*

Previously we reported that plant‐expressed *ΔspAvr2* complements virulence of a *FolΔAvr2* knockout, confirming that the protein is functional and acts inside the cell (Di *et al*., 2016). The Avr2^R45H^ variant carries a single amino acid change that does not affect its virulence function but allows the protein to evade I‐2‐mediated recognition (Houterman *et al*., 2009). To determine whether *ΔspAvr2* ^*R45H*^ could also complement the virulence defect of a *FolΔAvr2* strain, 10‐d‐old seedlings of wild‐type (WT) and two independent *ΔspAvr2* ^*R45H*^ expressing tomato lines (*ΔspAvr2* ^*R45H*^ *‐1* and *ΔspAvr2* ^*R45H*^ *‐11*) were inoculated with water (mock), WT Fusarium (*Fol007*) or the *FolΔAvr2* strain. As observed before (Houterman *et al*., 2007), Moneymaker plants inoculated with *Fol007* showed severe disease symptoms such as wilting and stunting. The *FolΔAvr2* strain is less pathogenic as shown by the increased vigor of the plants along with higher weights and lower disease indexes as compared to *Fol007* inoculation (Fig. S1). We found that disease symptoms of *FolΔAvr2*‐infected *ΔspAvr2* ^*R45H*^ plants were as severe as tomato plants infected with *Fol007* (Fig. S1). The regain of full pathogenicity of the *Fol Avr2* knockout strain on the *ΔspAvr2* ^*R45H*^ lines shows that *ΔspAvr2* ^*R45H*^ effectively complements fungal virulence.

**Figure 1:**
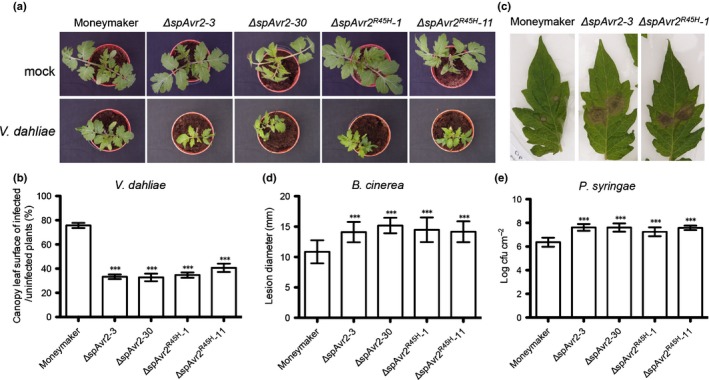
*ΔspAvr2* and *ΔspAvr2* ^*R45H*^ transgenic tomato plants show enhanced susceptibility to *Verticillium dahliae*,* Botrytis cinerea* and *Pseudomonas syringae*. (a) Representative pictures of mock (upper row) and race 1 JR2 (bottom row) inoculated Moneymaker, and *ΔspAvr2* and *ΔspAvr2* ^*R45H*^ transgenic tomato plants at 21 d post inoculation (dpi). (b) As a measure for disease severity, leaf canopy surface of inoculated plants was measured. (c) Disease symptoms of *B. cinerea* on tomato leaves. (d) Lesion development of *B. cinerea* on tomato leaves assessed at 3 dpi by determining the average lesion diameter on 10 leaves from three plants each. (e) Bacterial growth assays on tomato plants inoculated with *P. syringae* by syringe infiltration. Bacterial populations were measured at 3 dpi. Bars represents means ± SD. All experiments were repeated at least twice with similar results (***, *P *<* *0.001; one‐way ANOVA).

Previously we reported that *ΔspAvr2* expression in tomato promotes growth of the xylem‐infecting fungus *V. dahliae* resulting in enhanced disease symptoms (Di *et al*., 2016). To assess whether the I‐2‐recognition evading *ΔspAvr2* ^*R45H*^ variant also enhances susceptibility, *ΔspAvr2* and *ΔspAvr2* ^*R45H*^ tomato plants were inoculated with spores of *V. dahliae* strain JR2. Besides WT Moneymaker, two independent *ΔspAvr2*‐expressing tomato lines (*ΔspAvr2‐3* and *ΔspAvr2‐30*) and two *ΔspAvr2* ^*R45H*^‐expressing tomato lines (*ΔspAvr2* ^*R45H*^ *‐1* and *ΔspAvr2* ^*R45H*^ *‐11*) were tested. Typical symptoms of Verticillium wilt disease involve stunting, chlorosis, necrosis and vascular browning. Therefore, the canopy surface of inoculated plants was measured to quantify disease severity (Fradin *et al*., 2009). *Verticillium dahliae*‐inoculated Moneymaker plants showed moderate stunting when compared with mock‐inoculated plants (Fig. 1a). However, both *ΔspAvr2* and *ΔspAvr2* ^*R45H*^ plants appeared to be hypersusceptible to *V. dahliae* as they were much smaller in stature, and showed a significant reduction in canopy surface, as compared to the inoculated Moneymaker control plants (Fig. 1a,b). Overall, these data show that the *ΔspAvr2* and *ΔspAvr2* ^*R45H*^ plants are hypersusceptible towards *V. dahliae* as depicted by their enhanced fungal colonization (Di *et al*., 2016) and increased disease symptoms, indicative of an identical virulence activity for the Avr2 variants.

*Botrytis cinerea* is a necrotrophic plant pathogenic fungus that can infect many plant species. The infection process includes penetration of the host tissue and killing of the host cells, followed by lesion expansion, tissue maceration and sporulation (van Kan, 2006). To test whether Avr2 increases susceptibility to *B. cinerea*, detached leaves of 5‐wk‐old tomato plants were inoculated with a droplet of a conidial suspension of *B. cinerea* strain B05.10 (Fig. 1c). In *ΔspAvr2* and *ΔspAvr2* ^*R45H*^ tomato leaves, *B. cinerea* produced significantly larger lesions compared to those on the WT Moneymaker at 3 dpi (Fig. 1d). These data show that Avr2 enhances the susceptibility of tomato to infection with *B. cinerea*.

In order to determine whether the virulence‐promoting activity of Avr2 extends to pathogens other than the fungal pathogens tested above, its ability to enhance virulence to a bacterial pathogen, *Pst*, the causative agent of bacterial speck disease, was assessed. To monitor progress of disease development, 4‐wk‐old tomato plants were syringe‐infiltrated with *Pst* and leaf discs were collected from the infiltrated areas at 3 dpi. A significant increase in bacterial growth was observed (> 1 log cfu cm^−2^) in the *ΔspAvr2* and *ΔspAvr2* ^*R45H*^ lines as compared with Moneymaker (Fig. 1e). This demonstrates that expression of *ΔspAvr2* and *ΔspAvr2* ^*R45H*^ can increase susceptibility of tomato plants to bacterial as well as fungal pathogens.

### Avr2 induces a mild dwarfing phenotype and delayed germination in Arabidopsis

**Figure 2:**
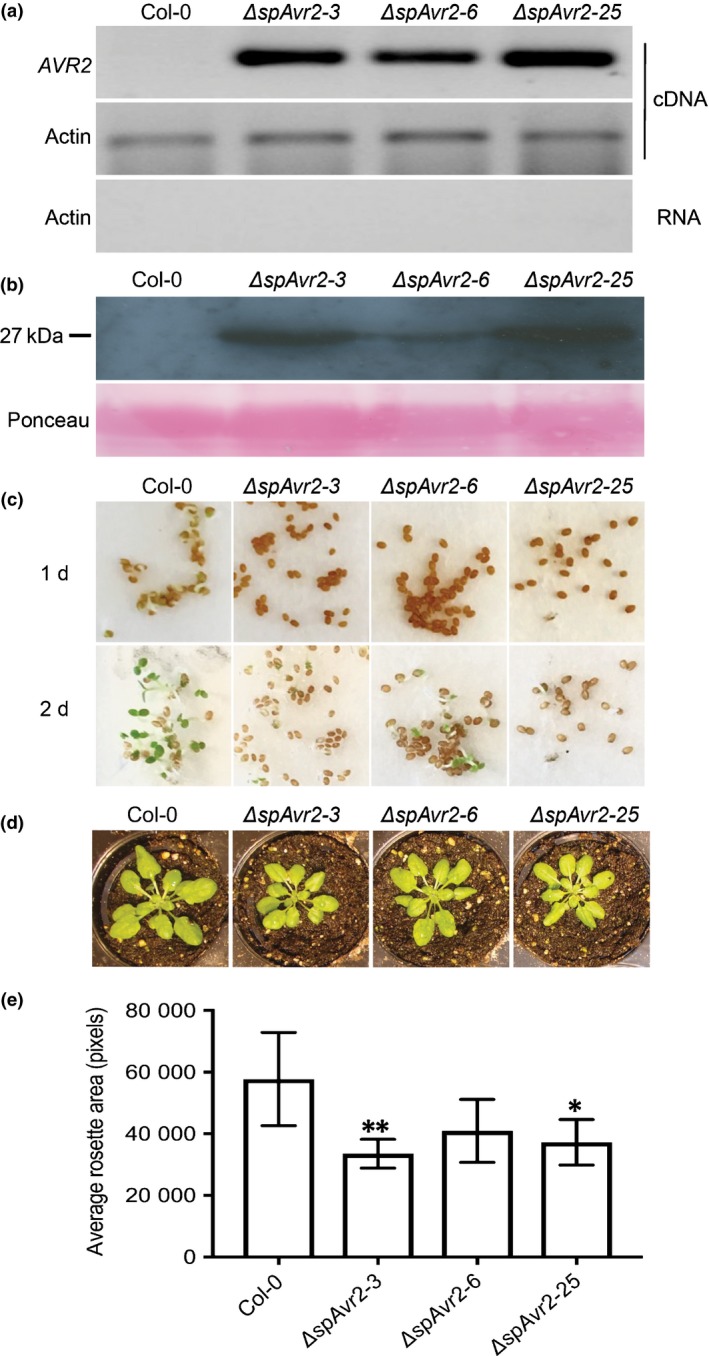
*ΔspAvr2* transgenic Arabidopsis plants show delayed germination and a dwarfing phenotype. (a) Reverse transcription polymerase chain reaction RT‐PCR of RNA isolated from leaves of 10‐d‐old plants using *AVR2* (upper panel) or Actin‐specific primers (lower panels). (b) Western blot of leaf total protein extracts probed with an Avr2 antibody. Ponceau S staining shows equal protein loading (lower panel). (c) Germination of wild‐type Col‐0 and *dspAvr2* transgenic Arabidopsis 1 d and 2 d after vernalization. One representative example of > 5 assays is shown. (d) Representative picture showing 4‐wk‐old Col‐0 and *ΔspAvr2* transgenic Arabidopsis plants. (e) Quantification of rosette area of 4‐wk‐old plants, graph shows mean ± SD. Ten plants/line were analyzed (*, *P *<* *0.05; **, *P *<* *0.01; one‐way ANOVA).

In order to test whether Avr2 also confers hypersusceptibility to *A. thaliana*, which may indicate the presence of an evolutionary conserved effector target, Col‐0 plants were transformed with the *ΔspAvr2* construct. The produced Avr2 protein carries at its Carboxy‐terminus an HA‐SBP tag to facilitate detection. Detection of *ΔspAvr2* expression and accumulation of the protein was assessed (respectively) by RT‐PCR (Fig. 2a) and Western blot analysis using an Avr2 antibody (Fig. 2b). Three independent *ΔspAvr2* transgenic T3 homozygous lines, *ΔspAvr2‐3*,* ΔspAvr2‐6* and *ΔspAvr2‐25,* showing clearly detectable *ΔspAvr2* expression, were kept for further analyses. As determined by Western blot analysis, the independent *ΔspAvr2* transgenic lines varied in their levels of Avr2 accumulation (Fig. 2b). Compared to Rubisco, which was detected by Ponceau staining and serves as a loading control, the *ΔspAvr2‐25* line accumulated higher amounts of ΔspAvr2 protein than *ΔspAvr2‐3* whereas line *ΔspAvr2‐6* showed the lowest Avr2 abundance.

Compared to WT Col‐0, all three *ΔspAvr2* transgenic Arabidopsis lines showed a delay in germination of *c*. 1 d (Fig. 2c). When grown under standard short day conditions, also a mild stunting phenotype was observed in all three *ΔspAvr2* Arabidopsis lines (Fig. 2d). To quantify the reduction of growth, the rosette leaf area of Col‐0 and *ΔspAvr2* transgenic plants was measured 28 d after sowing the seeds in soil (Fig. 2e). A significant reduction of rosette area was observed in two of the three *ΔspAvr2* plant lines in comparison to WT Col‐0. To conclude, high expression of *ΔspAvr2* results in a delay in Arabidopsis seed germination and plantlets having reduced rosette sizes.

### Avr2 enhances susceptibility of Arabidopsis to *F. oxysporum*,* V. dahliae* and to the bacterial pathogen *Pst* DC3000

**Figure 3:**
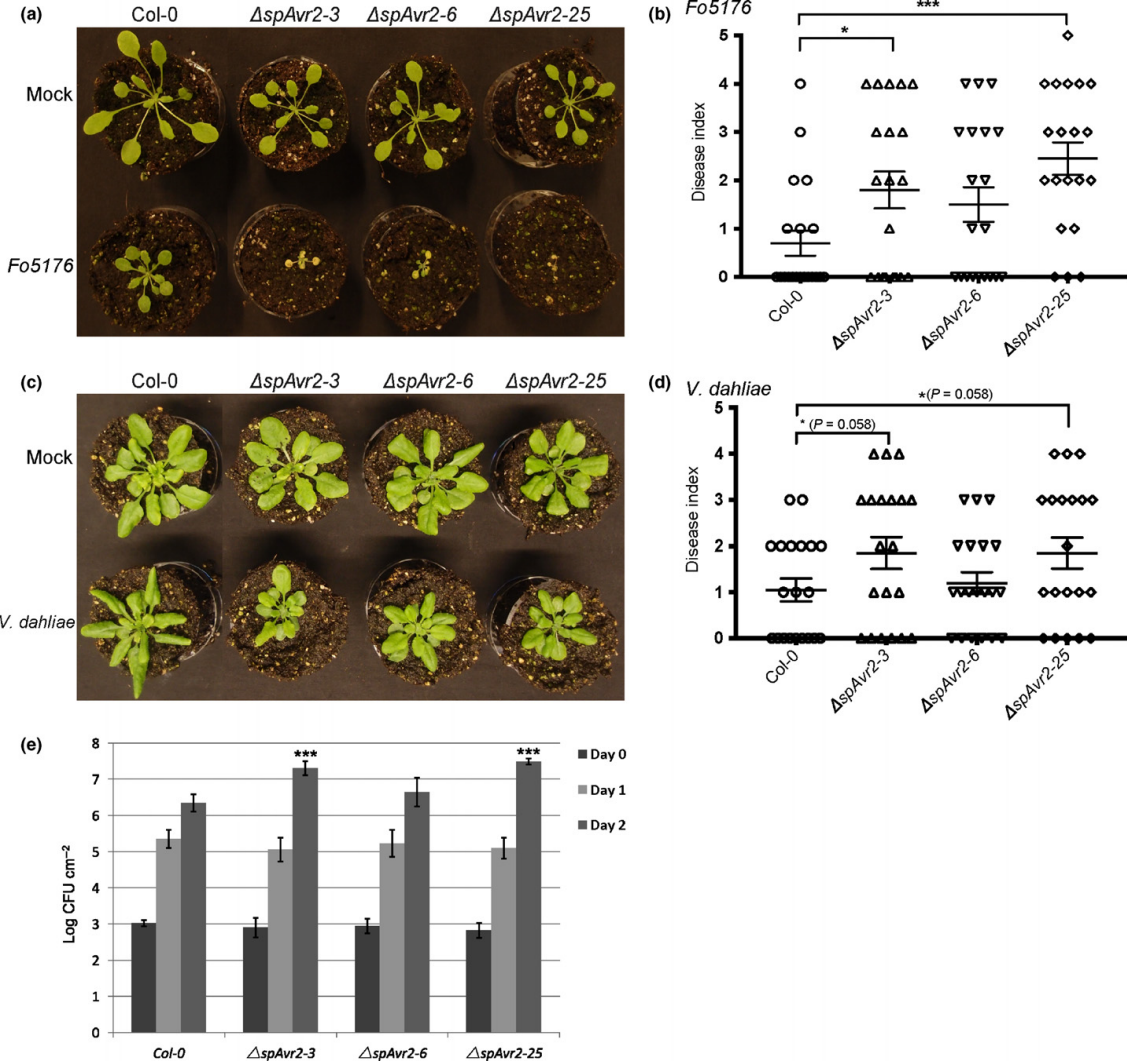
*ΔspAvr2* transgenic Arabidopsis lines exhibit increased susceptibility to *Fo5176, Verticillium dahliae* and *Pseudomonas syringae* pv. *tomato* (*Pst*) DC3000. (a) *Fo5176* bioassay on 14‐d‐old plants. Photographs of representative plants 10 d after mock (upper row) or *Fo5176* inoculation (lower row). The result from one representative repeat from three independent biological repeats is shown. (b) Disease index 10 d post inoculation (dpi) after *Fo5176* inoculation. Bars indicate ± SD of 20 replicates. *, *P *<* *0.05; **, *P *<* *0.01; ***, *P *<* *0.001 using one‐way ANOVA test. (c) *V. dahliae* bioassay on 14‐d‐old plants, representative photographs show disease development 21 dpi with mock (upper row) or fungus (lower row). (d) Disease index 21 dpi after *V. dahliae* inoculation. Bars indicate ± SD of 20 replicates. The result from one representative repeat from three independent biological repeats is shown, one‐way ANOVA. (e) *Pst* DC3000 was infiltrated into leaves of 5‐wk‐old Arabidopsis plants. Bacterial titers were measured at 0, 1, and 2 dpi (two leaf discs per replicate, error bars indicate standard deviation of four plants/line). Three biological replicates were performed and representative data from one experiment is shown. (***, *P *<* *0.001; *t*‐test).

In order to assess whether Avr2 increases disease susceptibility in Arabidopsis, bioassays were performed using different pathogens. First, the susceptibility of *ΔspAvr2* transgenic Arabidopsis plants to *F. oxysporum* was assessed. Bioassays were performed using Col‐0 WT and the three independent *ΔspAvr2* lines. Fourteen‐day‐old seedlings were root‐dip inoculated with the Arabidopsis‐infecting *F. oxysporum* strain *Fo5176*, a strain not carrying any Avr2 homologs (Thatcher *et al*., 2012). Disease index was scored at 10 dpi on a scale from 0 to 5, in which 0 means no disease symptoms and 5 indicates dead plants (Gawehns *et al*., 2014). A significant increase (*P *<* *0.05) in disease symptoms was observed in two of the three *ΔspAvr2* lines (Fig. 3a,b). In addition, the *ΔspAvr2‐25* line that showed highest ΔspAvr2 accumulation (Fig. 2a) also showed the highest disease index.

In order to monitor susceptibility to Verticillium wilt disease, 14‐d‐old Arabidopsis seedlings were inoculated with *V. dahliae* spores and disease index was scored at 21 dpi (Fig. 3c). To quantify disease symptoms, the amount of chlorosis was scored (Gawehns *et al*., 2014). Compared to Col‐0, a nearly significant (*P *=* *0.058) increase in disease symptoms was observed in two of the three *ΔspAvr2* lines (*ΔspAvr2‐3* and *ΔspAvr2‐*25). The *ΔspAvr2‐6* line showed disease symptoms indistinguishable from that of WT plants (Fig. 3d).

Next, it was examined whether expression of *Avr2* affects susceptibility to the bacterial pathogen *Pst* DC3000. Inoculation was done on Col‐0 WT plants and the three independent *ΔspAvr2* lines. Five‐week‐old Arabidopsis plants were syringe‐infiltrated with a *Pst* DC3000 culture having an OD_600_ of 0.0005. Leaf discs were collected from the infiltrated leaf areas at 0, 1 and 2 dpi and bacterial growth was determined. At 0 dpi a similar bacterial titer was observed in all infiltrated plants showing an even inoculation (Fig. 3e), but at 2 dpi a significant increase in bacterial growth of approx. 1 log difference (*P *<* *0.001) was observed in two of the three *ΔspAvr2* lines (*ΔspAvr2‐3* and *ΔspAvr2‐*25) in comparison to Col‐0 plants (Fig. 3e). No significant differences in bacterial growth were observed between Col‐0 plants and *ΔspAvr2‐6* plants (Fig. 3e). Based on these data we conclude that elevated accumulation of ΔspAvr2 enhances disease susceptibility to the bacterial pathogen *Pst* DC3000 and the fungi *V. dahliae* JR2 and *F. oxysporum Fo5176*.

### ROS production and callose deposition are reduced in *ΔspAvr2* tomato

**Figure 4:**
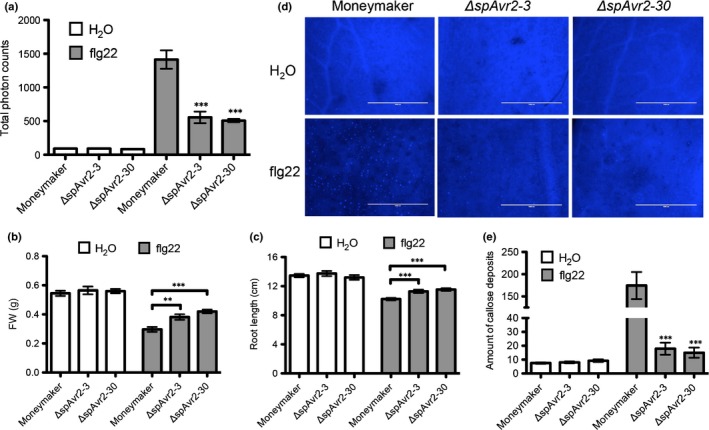
Physiological changes upon flagellin22 (flg22) treatment of wild‐type and *ΔspAvr2* tomato. (a) Flg22‐induced oxidative burst measured over a period of 50 min. Reactive oxygen species (ROS) were quantified by measuring the total photon counts emitted over time using a luminol‐chemiluminescence assay; (b) FW and (c) root length inhibition of tomato plants in liquid Murashige & Skoog media containing either H_2_O or flg22 (100 nM) was analyzed 7 d post inoculation. (d) Microscopic comparison of callose deposits after flg22 infiltration and staining with aniline blue. (e) Total number of callose deposits per field of view is depicted. Error bars represent standard error of 10 biological replicates (***, *P *<* *0.001; **, *P *<* *0.01; one‐way ANOVA).

Because expression of *ΔspAvr2* promotes hypersusceptibility of tomato and Arabidopsis to a variety of microbial pathogens, we hypothesized that Avr2 might interfere with basal host immune responses. To test whether Avr2 interferes with pattern recognition receptor (PRR)‐mediated signaling, we compared the flg22‐induced ROS burst in Moneymaker and two independent *ΔspAvr2* tomato lines using a luminol/peroxidase‐based assay. Leaf discs of 4‐wk‐old tomato plants were treated with either flg22 or water. Leaf discs incubated with water did not produce a significant ROS burst (Fig. 4a). As expected, flg22 treatment of leaf discs derived from Moneymaker plants generated a ROS burst, with a total of *c*. 1500 photon counts over a 50‐min period. Compared to the WT plants, both *ΔspAvr2* lines showed a severely reduced ROS accumulation, with only *c*. 500 photon counts emitted (Fig. 4a). These data show that the flg22‐trigged ROS burst is suppressed in *ΔspAvr2* plants, suggesting that FLS2‐mediated signaling is compromised in these plants.

### Avr2 represses growth inhibition of tomato seedlings induced by flg22 treatment

In order to further investigate the role of Avr2 as a suppressor of PRR‐mediated signaling, growth inhibition induced by flg22 treatment was monitored in *ΔspAvr2* transgenic tomato seedlings (Pfund *et al*., 2004). FW and root length were recorded 10 d after transferring to liquid media. Significant growth inhibition was observed in Moneymaker upon flg22 treatment; both plant FW and root length were reduced (Fig. 4b,c). Although growth was also inhibited in *ΔspAvr2* plants following flg22 treatment, the reduction was significantly less than that of WT plants (Fig. 4b,c). Additionally, we tested the flg22‐treated Moneymaker and *ΔspAvr2* plants for callose deposition by staining the cotyledons with aniline blue and determining the number of UV‐fluorescent deposits using fluorescence microscopy. The amount of callose depositions was severely reduced in *ΔspAvr2* plants as compared to Moneymaker following flg22 treatment (Fig. 4d,e). The observed attenuation of ROS production, reduction in growth inhibition and decreased callose depositions indicate that Avr2 is capable of inhibiting FLS2‐mediated immune responses in tomato plants.

### Avr2 suppresses flg22‐induced ROS burst and callose production in Arabidopsis

**Figure 5:**
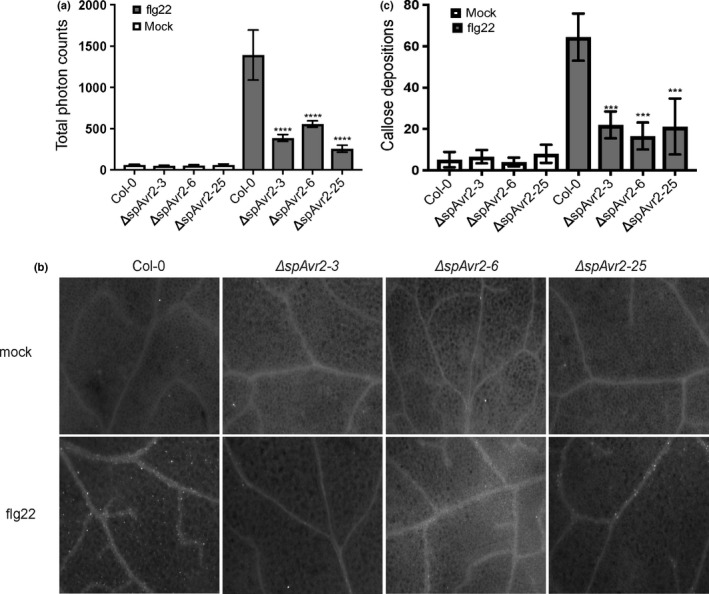
Physiological changes upon flagellin22 (flg22) treatment of wild‐type and *ΔspAvr2* Arabidopsis. (a) Flg22‐induced reactive oxygen species (ROS) production in Arabidopsis. Leaf discs of 5‐wk‐old Arabidopsis were treated with either mock or with 100 nM of flg22. ROS was quantified by measuring the total photon counts over time following flg22 treatment. Error bars indicate ± SD of three replicates. (****, *P *<* *0.0001; one‐way ANOVA). (b) Callose depositions quantified 24 h post mock or flg22 treatment. Representative images showing callose deposition in Arabidopsis leaves as observed by fluorescence microscopy. (c) Callose depositions were significantly induced in Col‐0 upon flg22 treatment but were suppressed in *ΔspAvr2* Arabidopsis. Quantification of callose deposits was measured by ImageJ and each data point represents the mean of six replicates. Error bars indicate ± SD (***, *P *<* *0.001; one‐way ANOVA).

In order to investigate whether Avr2 also suppresses FLS2‐mediated responses in Arabidopsis, ROS production and callose deposition in the *ΔspAvr2* transgenic lines following flg22 treatment was monitored. Leaf discs isolated from Col‐0 control plants and the three independent *ΔspAvr2* lines were treated with either 100 nM flg22 or water as negative control. Luminescence was quantified over a 40‐min period after flg22 or water application. The accumulative ROS burst of the flg22‐treated Col‐0 control added up to a total of *c*. 1400 photon counts in 40 min. Compared to WT Arabidopsis, all *ΔspAvr2* carrying lines showed a significant reduction of flg22 induced ROS formation (Fig. 5a).

Callose deposition following flg22 treatment was visualized using an aniline blue stain 24 h after infiltration in Arabidopsis leaves (Fig. 5b). Compared to nontreated plants a strong increase of callose deposition was observed in treated WT Arabidopsis leaves to *c*. 65 dots per image (Fig. 5c). In comparison to WT Arabidopsis, significantly fewer callose depositions were observed in *ΔspAvr2‐3*,* ΔspAvr2‐6* and *ΔspAvr2‐*25 leaves after flg22 treatment, with an average of < 30 deposits per image. In conclusion, ΔspAvr2 suppresses flg22‐induced immune responses in Arabidopsis, as both the ROS burst and callose depositions are attenuated.

### The crystal structure of Avr2 reveals a β‐sandwich fold and surface presentation of residues altered in immunity evading race3 Avr2 variants

**Figure 6:**
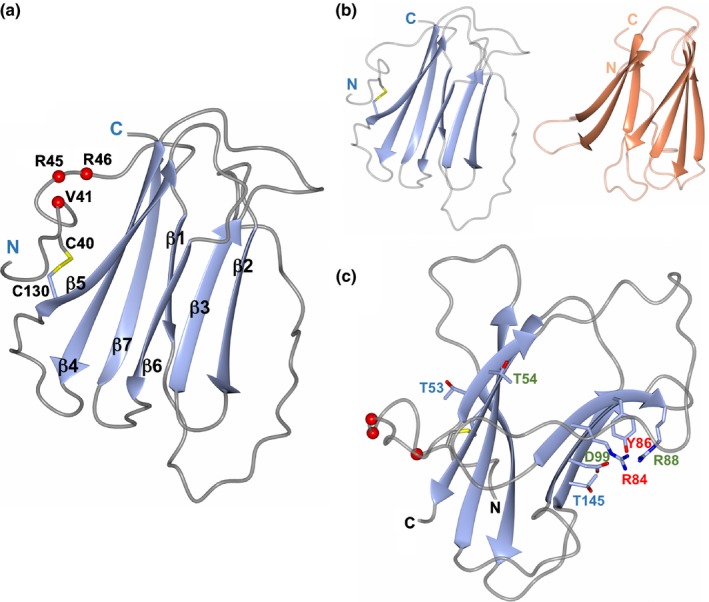
The crystal structure of Avr2. (a) Avr2 comprises a β‐sandwich fold with two antiparallel β‐sheets. The strands are numbered sequentially from β1 to β7 with sheet I composed of strands β1, β7, β4 and β5, and sheet II containing strands β2, β3, and β6. The disulphide bond between residues C40 and C130 is shown in yellow with the cysteine side chains in stick representation. The positions of variants in *Fol* race 3 strains that evade recognition by I2 are shown as red spheres. (b) Side‐by‐side representations of the structures of Avr2 (left) and the ToxA overlay (right, orange), showing the similar overall protein folds. The orientation of Avr2 in panels (a) and (b) is the same. (c) The positions of the Avr2 mutants generated in this study are shown with side chains in stick representation. Those labeled in red are mutants that affected protein accumulation in plants, those in green are mutants that behave as wild‐type (for D99E, D99A resulted in unstable protein), and those in blue affected the putative virulence function without altering I2‐mediated recognition. The N‐ and C‐termini are labeled in each panel. Structure figures were prepared with CCP4MG (McNicholas *et al*., 2011).

In order to advance our understanding of how Avr2 functions at the molecular level, we determined the crystal structure of this protein to atomic resolution (1.1 Å). Avr2 adopts a β‐sandwich fold formed from two antiparallel β‐sheets (Fig. 6a). The first β‐sheet comprises strands β1, β7, β4 and β5, and the second sheet contains strands β2, β3 and β6 (Fig. 6a). The electron density reveals a disulphide bond between Cys40 and Cys130. Cys40 is located close to the N‐terminus of the protein whereas Cys130 maps at the start of β5. The position of these residues within the context of the fold suggests a role in stabilizing the overall structure of the protein, and specifically to anchor the N‐terminus to the core of the β‐sandwich. The anchoring of this region via a disulphide bond is of interest as *Fol* race 3 Avr2 variants, which enable evasion of immunity on infection of tomato (Avr2^V41M^, Avr2^R45H^, Avr2^R46P^ and Avr2^T50−^) (Houterman *et al*., 2009; Chellappan *et al*., 2017), are all located on a loop structure between Cys40 and the start of β1 (Fig. 6a). In contrast to previous *in planta* data (Ma *et al*., 2013), analysis of protein interfaces in the crystals of Avr2 (using PDBePISA; Krissinel & Henrick, 2007) suggests that the effector is unlikely to oligomerize in solution.

### Avr2 shares structural homology with ToxA and TRAF‐domain proteins

Searches using DaliLite (Holm & Rosenstrom, 2010) and PDBeFold (Krissinel & Henrick, 2004) reveal that the closest structural homolog to Avr2 is ToxA, an effector from the fungal wheat pathogen *Pyrenophora tritici‐repentis* (Sarma *et al*., 2005; Manning *et al*., 2008) (Fig. 6b). ToxA induces necrosis and disease susceptibility dependent on the host protein Tsn1 (a Nucleotide binding domain, Leucine rich repeat Receptor (NLR)‐like protein, with an integrated kinase domain; Faris *et al*., 2010). However, the ToxA structure reveals little insight about the molecular mechanism of action. Like Avr2, ToxA is stabilized by the presence of a disulphide bond between Cysteine residues towards the beginning and the end of the structural domain. An Arg‐Gly‐Asp (RGD)‐motif has been shown to be required for ToxA entry into host plant cells by its proposed interaction with integrin‐like receptor proteins (Manning *et al*., 2008), however, this motif is not conserved in Avr2.

Avr2 only shares 5% sequence identity with ToxA (revealing no significant sequence similarity), but the structures overlay with a root mean square deviation (r.m.s.d.) of 2.8 Å over 86 residues (as reported by DaliLite, Fig. 6b). Avr2 also shows structural homology to Tumor necrosis factor Receptor Associated Factor (TRAF) domains although, as for ToxA, pairwise sequence identities are typically < 5%. Despite this, Avr2 overlays on the TRAF‐domains of Speckle‐type POZ (SPOP) protein (PDB: 4O1V; Li *et al*., 2014a), with TRAF6 (PDB: 1LB5 (Ye *et al*., 2002)) and with SIAH1 (PDB: 4X3G; Zhang *et al*., 2017), with r.m.s.d.s of 3.5 Å over 97 residues, 3.2 Å over 95 residues and 2.8 Å over 86 residues, respectively (Figs S2 and S3). TRAFs are cytoplasmic adaptor proteins involved in cell signaling cascades in mammals, including roles in PRR‐ and NLR‐mediated signaling (Xie, 2013). Plants also contain TRAFs. Recently, two Arabidopsis TRAF domain‐containing proteins were shown to regulate NLR turnover, potentially *via* interacting with E3 ligase complexes and modulating ubiquitination (Huang *et al*., 2016).

### Site‐directed mutagenesis of Avr2 allows uncoupling of virulence from recognition

Using existing TRAF‐domain structures in complex with peptide ligands to identify putative functionally relevant sites, we designed a set of mutants in Avr2 (see Methods S1 and Fig. S4). Our strategy was to replace small and/or noncharged residues for larger charged ones, or *vice versa*. We generated a total of nine variants in the ΔspAvr2 background for *in planta* expression: Avr2^T53R^, Avr2^T54R^, Avr2^R84A^, Avr2^Y86A^, Avr2^R88A^, Avr2^D99E and D99A^, and Avr2^T145E and T145K^. Interestingly, residues Avr2^T53^ and Avr2^T54^ are located on the same face of the protein as the N‐terminal loop harboring the mutations in *Fol* race 3 variants that evade I‐2‐mediated recognition (Fig. 6c). All other mutations map at the opposite face of the protein, distal from this N‐terminal loop (Fig. 6c).

**Figure 7:**
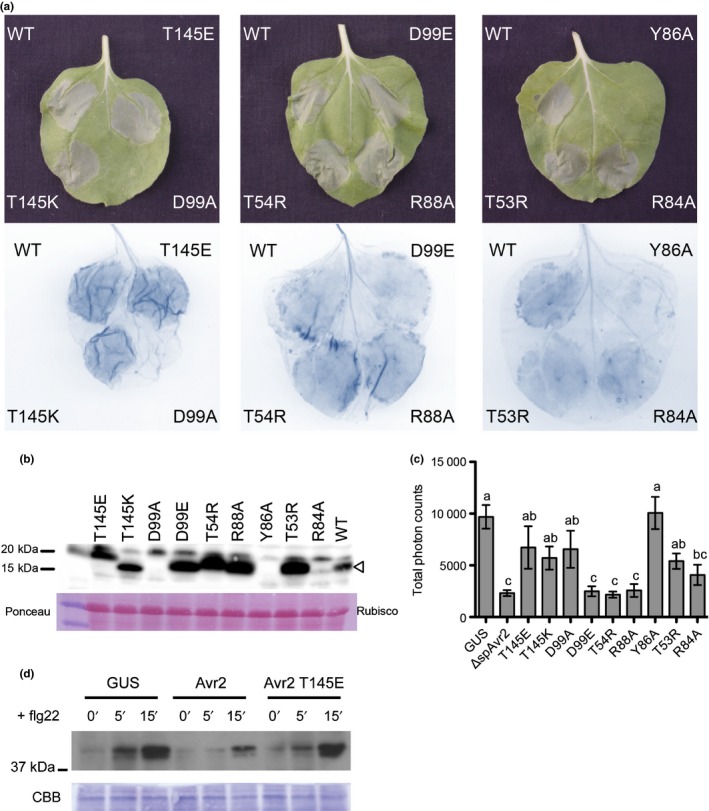
Assessment of I‐2‐mediated hypersensitive response (HR) in *Nicotiana benthamiana* following co‐expression of *I‐2* with *ΔspAvr2* mutants. (a) A transient expression assay in *N. benthamiana* leaves using *Agrobacterium tumefaciens* co‐expressing the *ΔspAvr2* mutants with the *I‐2* gene to show I‐2‐mediated cell death (top panel). Cell death is visualized by trypan blue staining of the infiltrated leaves (bottom panel). (b) Western blot shows protein accumulation in the various Avr2 variants (Avr2 is indicated with an arrowhead). (c) Leaves of *N. benthamiana* transiently expressing *ΔspAvr2* and its variants after agro‐infiltration were treated with the flg22 elicitor and generation of reactive oxygen species (ROS) was monitored. Some ΔspAvr2 mutants (Avr2^T145E^, Avr2^T145K^, Avr2^D99A^, Avr2^Y86A^ and Avr2^T53R^) lost their capacity to repress ROS production after flg22 treatment like the GUS control (one‐way ANOVA *P *<* *0.05, different letters indicate statistically significant differences, error bar mean ± SEM). (d) Leaves of *N. benthamiana* transiently expressing GUS, Δsp*Avr2* and the *ΔspAvr2* ^*T145E*^ variant were treated with flg22 for 0, 5 and 15 min and presence of phosphorylated MAP kinases was monitored by Western blotting. Coomassie staining of the blot is shown as loading control. WT, wild‐type.

First, we assessed whether these mutations affected I‐2‐mediated recognition. For this, the mutants were co‐expressed with *I‐2* in *Nicotiana benthamiana* (Fig. 7a). As previously shown, WT Avr2, but not the *Fol* race 3 variants Avr2^V41M^, Avr2^R45H^ and Avr2^R46P^ triggered a hypersensitive response (HR) (Houterman *et al*., 2009). Two of the nine *ΔspAvr2* mutants tested, ΔspAvr2^D99A^ and ΔspAvr2^Y86A^, failed to trigger an HR, whereas the others all retained I‐2‐mediated recognition (Fig. 7a). To confirm protein accumulation, Western blot analysis using Avr2 specific antibodies was performed. This revealed similar or slightly elevated accumulation of the mutants ΔspAvr2^T145E^, ΔspAvr2^T145K^, ΔspAvr2^D99E^, ΔspAvr2^T54R^, ΔspAvr2^R88A^ and ΔspAvr2^T53R^ following Agro‐mediated expression compared to WT (Fig. 7b). A strongly reduced accumulation of ΔspAvr2^R84A^ was observed, which, however, is apparently sufficient to trigger I‐2 activation (Fig. 7a). The difference in the position of the ΔspAvr2^T145E^ mutant on the blot is likely due to the change in charge of the protein. We observed that the ΔspAvr2^D99A^ and ΔspAvr2^Y86A^ variants did not accumulate to detectable levels, which is consistent with their inability to trigger I‐2‐mediated recognition. This suggests that these mutations compromise protein stability.

In order to determine whether the Avr2 mutants have preserved their virulence function, their ability to suppress the flg22‐induced ROS burst was monitored. For this, the *ΔspAvr2* variants were transiently expressed in *N. benthamiana* leaves. Transient expression of *ΔspAvr2* severely reduces ROS accumulation compared to the *GUS* control in *N. benthamiana* (Fig. 7c), consistent with the observations in tomato and Arabidopsis (Figs 4a, 5a). In line with the Western blot analysis, the unstable ΔspAvr2^D99A^ and ΔspAvr2^Y86A^ mutants did not suppress ROS formation following flg22 treatment. Mutants ΔspAvr2^D99E^, ΔspAvr2^T54R^ ΔspAvr2^R84A^, and ΔspAvr2^R88A^, which retained I‐2‐mediated recognition, still reduced flg22‐induced ROS burst, similar to the WT protein. In mutants ΔspAvr2^T145K/E^ and ΔspAvr2^T53R^, however, the virulence activity was uncoupled from I‐2‐mediated recognition. The virulence activity and recognition of each ΔspAvr2 variants are summarized in Table 2.

**Table 2:** Summary of virulence and I‐2 recognition activities of *Fusarium oxysporum* ΔspAvr2 mutants in *Nicotiana benthamiana*

Mutation	I‐2‐mediated HR	Protein accumulation	Flg22‐induced ROS suppression
Thr145Glu	+	+	−
Thr145Lys	+	+	−
Asp99Ala	−	−	−
Asp99Glu	+	+	+
Thr54Arg	+	+	+
Arg88Ala	+	+	+
Tyr86Ala	−	−	−
Thr53Arg	+	+	−
Arg84Ala	+	+/−	+
Wild‐type Avr2	+	+	+

a HR, hypersensitive response; ROS, reactive oxygen species.

Next, we wanted to test whether Avr2 mutants are impaired in suppression of FLS2 responses other than the ROS burst. For this we assessed the activation of MAP kinases in *N. benthamiana* leaves transiently expressing *GUS*,* ∆spAvr2* or the *∆spAvr2* ^*T145E*^ variant. Leaf discs were exposed to flg22 for 0, 5 and 15 min, and the accumulation of phosphorylated MAPKs was determined by Western blotting. As previously reported for *Arabidopsis* and *N. benthamiana*, we detect a specific signal *c*. 42 kDa after 5 min flg22 treatment, which was further increased upon 15 min treatment (Saijo *et al*., 2009; Segonzac *et al*., 2011)(Fig. 7d). This signal was markedly reduced, albeit detectable, in leaves expressing *∆spAvr2*, suggesting that WT Avr2 suppresses FLS2‐induced activation of MAPKs. In line with its reduced effect on the ROS burst, the *∆spAvr2* ^*T145E*^ variant showed impaired suppression of MAPK activation.

### A nuclear localization of Avr2 is not required for its PTI‐suppressing activity

**Figure 8:**
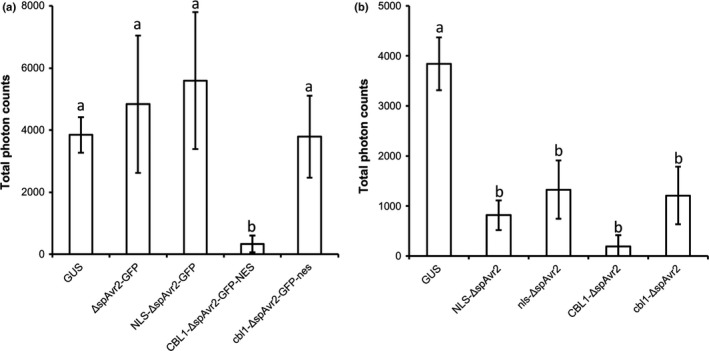
A nuclear localization of Avr2 is not required to suppress flg22‐induced reactive oxygen species (ROS) production in *Nicotiana benthamiana*. (a) flg22‐triggered ROS production in *N. benthamiana* leaves transiently expressing *ΔspAvr2‐GFP* variants targeted to different subcellular compartments. (b) flg22‐triggered ROS production in *N. benthamiana* leaves transiently expressing nontagged Δsp*Avr2* variants targeted to the nucleus and plasma membrane, respectively. The ΔspAvr2 variants do not carry a C‐terminal GFP tag. Different letters indicate statistically significant difference (one‐way‐ANOVA;* P *<* *0.01, error bars denote ± SD).

It was previously established that a nuclear localization of Avr2 is required for recognition by I‐2 when both proteins are transiently expressed in *N. benthamiana* (Ma *et al*., 2013). Specifically, fusion of a nuclear targeting signal to Avr2 resulted in a predominantly nuclear accumulation without reducing its recognition by I‐2. By contrast, fusing a myristoylation motif to Avr2 N‐terminally resulted in its capture at the plasma membrane (PM) and prevented activation of I‐2 (Ma *et al*., 2013). We used these published constructs to test where Avr2 is acting in the cell to suppress flg22‐triggered ROS burst. Surprisingly, we observed that expression of C‐terminally GFP‐tagged ∆spAvr2 did not result in a significant ROS suppression, indicating that the GFP tag interferes with the virulence activity of Avr2. Likewise, ∆spAvr2‐GFP fused to a nuclear targeting signal, or to the mutated myristoylation motif, showed no significant suppression of flg22‐induced ROS burst either. By contrast, targeting Avr2‐GFP to the plasma membrane with a functional myristoylation motif resulted in robust suppression of the flg22‐triggered ROS burst (Fig. 8a). As, in agreement with previous studies (Ma *et al*., 2013), the Avr2‐GFP signal was not detected in the nucleus this demonstrates that the myristyolation motif successfully excluded Avr2 from the nucleus (Fig. S5). Together these data suggest that the PRR‐triggered immunity (PTI)‐suppressing function of Avr2 is executed at, or closely to, the PM. To further evaluate if targeting of Avr2 to the nucleus may also contribute to ROS suppression, we generated constructs in which non‐GFP tagged Avr2 was fused to a functional NLS, a mutated NLS (nls), a functional myristoylation motif and a mutated myristoylation motif. Expression of all four constructs in *N. benthamiana* leaves resulted in suppressed ROS burst when compared to a GUS control, indicating that either targeting of Avr2 to the nucleus was incomplete, or both a nuclear and a PM‐localized pool of Avr2 mediates PTI suppression.

## Discussion

Effector proteins modulate host physiology to support parasitism (Dodds & Rathjen, 2010). Many effectors suppress host immune responses, priming the host for susceptibility. We here reveal that the *Fusarium oxysporum* f.sp. *lycopersici* (*Fol*) effector protein Avr2 targets an evolutionarily conserved immune pathway (likely an early component in pattern recognition receptor (PRR)‐triggered immunity (PTI) signaling), as both *ΔspAvr2* transgenic tomato and Arabidopsis plants became hypersusceptible to pathogen attack. An intracellular PTI‐perturbing activity for this effector fits its intracellular recognition by I‐2 (Houterman *et al*., 2009), and its pathogen‐mediated uptake by the host (Di *et al*., 2016). Such activities correlate with those of bacterial and oomycete PTI‐targeting effectors (Fawke *et al*., 2015; Macho & Zipfel, 2015). Our structure/function analysis suggests that Avr2 may act as an adaptor protein to modulate cell‐signaling cascades.

Stable expression of *ΔspAvr2* in *Arabidopsis thaliana* and in tomato increases susceptibility to the fungal pathogens *F. oxysporum* and *Verticillium dahliae*, as well as to the bacterial pathogen *Pseudomonas syringae*. Interestingly, in Arabidopsis the extent of susceptibility was correlated to the accumulation level of ΔspAvr2 in the plant. The *ΔspAvr2‐6* line having the lowest Avr2 accumulation (Fig. 2a) did not exert increased susceptibility to *F. oxysporum*,* V. dahliae* or *P. syringae,* whereas *ΔspAvr2‐25* showing the highest Avr2 dose had the most severe disease symptoms to all tested pathogens. This dose‐dependency suggests that the virulence‐promoting properties of Avr2 may involve competition with a host factor for target‐binding, or that a threshold of activity is required to alter plant physiology to enable susceptibility.

Although the host target of Avr2 is unknown, the data here suggest that it is a factor involved in PRR‐mediated immunity signaling that is evolutionarily conserved in tomato, Arabidopsis and *N. benthamiana*. Although effector‐triggered immunity (ETI) and PTI share downstream signaling components (Dodds & Rathjen, 2010), we focused on PTI‐related outputs as it has been previously shown that ETI signaling is not compromised in the presence of Avr2 (Gawehns *et al*., 2014). We found that *ΔspAvr2* expression in Arabidopsis, tomato and *N. benthamiana* compromises classical PTI‐associated readouts (reactive oxygen species (ROS) burst, callose deposition, mitogen‐activated protein kinases (MAPK), activation and reduced growth) and leads to susceptibility to various pathogens. The broad‐spectrum susceptibility conferred by Avr2 suggests that it does not target a specific PRR, with a confined pattern recognition profile, but instead either a variety of PRRs or a conserved downstream component. It is formally also possible that Avr2 could affect PRR biogenesis or transport (Nekrasov *et al*., 2009). However, as we observe only a mild stunted phenotype in *ΔspAvr2* transgenic Arabidopsis plants (Fig. 2), and no apparent developmental phenotypes in *ΔspAvr2* transgenic tomato (Fig. 1), a role for general interference in trans‐membrane receptor function seems unlikely, as these receptor types are also involved in control of growth and development (Hecht *et al*., 2001).

The effects of Avr2 on PTI responses in Arabidopsis largely mimic the phenotypes of null mutants in kinases such as Botrytis‐induced kinase 1 (BIK1) and the related PBS1‐like kinase (PBLs) (Zhang *et al*., 2010). These kinases are themselves phosphorylated by an activated PRR–Brassinosteroid‐insensitive 1 (BRI1)‐Associated Receptor Kinase 1 (BAK1) complex following pathogen‐associated molecular pattern (PAMP) perception (Kadota *et al*., 2014; Li *et al*., 2014b). Phosphorylated BIK1 and PBL1 can directly phosphorylate the NADPH oxidase Respiratory Burst Oxidase Homologue Protein D (RBOHD) complex to activate ROS production. Furthermore, both kinases trigger a MAPK cascade (Couto & Zipfel, 2016); both readouts are suppressed in Avr2 plants. Intriguingly, in addition to being compromised in both ROS production and callose deposition, the *bik1* mutant has a dwarfing phenotype, another parallel with expression of *ΔspAvr2* in Arabidopsis (Veronese *et al*., 2006). Another potential target for Avr2 is the PRR co‐receptor BAK1. Whilst BAK1 is well established as having a role in PRR‐related signaling, it also functions as a co‐receptor for BRI1) (Li *et al*., 2002; Nam & Li, 2002). A *bri1* mutant exerts a dwarf phenotype similar to that of *ΔspAvr2* plants (Li & Chory, 1997). Hence, suppressing BAK1 activity will compromise not only PTI, but also plant development by impairing BRI1 function. The rice pathogen *Xanthomonas oryzae* targets BAK1 and its type III effector protein Xoo2875 interacts with BAK1, and its expression in rice results in a semi‐dwarf phenotype and a compromised PTI response (Yamaguchi *et al*., 2013). Taken together, the data are consistent with Avr2 targeting the PRR–BAK1–BIK1/PBL1 complex at the plasma membrane, thereby interfering with PTI signaling and plant growth. The localization of the complex at the plasma membrane (PM) corresponds with the site of action in the cell where Avr2 exerts its PTI‐suppressing activity, which is retained when tethered to the PM by a myristyolation motif. Future experiments could investigate whether Avr2 physically interacts with and/or alters the turnover of either BAK1, BIK1 or PBL1.

Natural variation in Avr2 in *Fol* race 3 isolates abolishes I‐2‐mediated recognition, but does not affect virulence (Houterman *et al*., 2009; Chellappan *et al*., 2017). The mechanism by which the NLR I‐2 recognizes Avr2, be it direct or indirect, is unknown. The crystal structure of Avr2 has revealed that *Fol* race 3 variants Avr2^V41M^, Avr2^R45H^, Avr2^R46P^ and Avr2^T50−^ are all within surface‐exposed residues that cluster on a loop, forming a distinct epitope (Fig. 6a). Notably, the residues that form this epitope are anchored in position by Cys40 forming a disulphide bound at one end (the far N‐terminus), and at the start of β1 (residue Ser51). Such an anchored extension is not observed in ToxA or other TRAF‐like proteins. We predict that molecular interactions involving this region are critical for I‐2‐mediated recognition and underlie the ability of the *Fol* race 3 variants to evade this.

A specific aim of our structure‐informed mutagenesis strategy was to identify mutations in Avr2 that result in a loss of virulence, but retain I‐2‐mediated recognition. This would show that the recognition of Avr2 by I‐2 is not reliant on its virulence activity, and the residues involved in these activities are not overlapping. Our screen identified two threonine residues in Avr2 (Avr2^T53^ and Avr2^T145^) that are required for virulence (suppression of PTI), but not for I‐2‐mediated recognition. Somewhat surprisingly, these two residues are not co‐localized in the structure and lie on opposite faces of the β‐sandwich. Avr2^T145^ is positioned within a putative functional site identified from comparison with the ligand‐bound structures of Speckle‐type POZ (SPOP) protein and TRAF6, and Avr2^T53^ is positioned within a putative functional site identified from comparison with the ligand‐bound structure of SIAH1. Interestingly, Avr2^T53^ is located close to the loop containing the residues Avr2^V41^, Avr2^R45^ and Avr2^R46^ that, when mutated, have given rise to evasion of *I‐2* resistance. Nevertheless, the data from these two mutants support a hypothesis that I‐2‐mediated recognition is a direct event and does not involve changes that Avr2 exerts on its virulence target in the cell. Besides direct recognition of Avr2 alone, recognition by I‐2 might be based on the proximity of two epitopes, one present on the loop region in Avr2, and the other present on the interacting host target. In this latter scenario, the Avr2^T53R^, Avr2^T145K^ and Avr2^T145E^ mutants retain the capacity to interact with the host target in a similar way as the wild‐type protein, but not exert the effect that promotes disease.

Structural homology searches revealed that Avr2 shares an overall β‐sandwich fold with the *Pyrenophora tritici‐repentis* effector ToxA and TRAF‐domain containing proteins, despite sharing no identifiable sequence similarity (no difference from chance). For some families of filamentous plant pathogens, the presence of shared structure in the absence of sequence conservation argues for a common evolutionary ancestor that has expanded by duplication, and diversified over time (Franceschetti *et al*., 2017). Overall shared structure, with limited or essentially no recognizable sequence identity, is an emerging theme in filamentous plant pathogen effectors (Wirthmueller *et al*., 2013). Such relationships are prevalent in phylogenetically related plant pathogens such as in the Peronosporales (Win *et al*., 2012) and *Pyricularia* (de Guillen *et al*., 2015) genera, but also have be identified in distantly related species (de Guillen *et al*., 2015). Given the lack of sequence identity, concluding that Avr2 shares a specific function with Tumor necrosis factor Receptor Associated Factor (TRAF)‐domain containing proteins (such as interfering with host ubiquitin ligase activity) is not possible. However, it is tempting to speculate that Avr2, in having this protein fold, may function (like other TRAFs) as an adapter unit, competing with host factors for target binding or binding to a host target and modulating its function. Whether functional requirements have steered convergent evolution, or whether these proteins share a common ancestor, requires future study.

Studies addressing the molecular mechanisms of how plant pathogen effectors modulate host cells for their own benefit, and how they are recognized by the plant innate immune system, remain a major focus of research in the plant/microbe interaction field. In recent years, identification of the host targets of effectors has enabled a deeper understanding of their activities and suggested new methods for engineering disease resistance (Gawehns *et al*., 2013). To resolve the molecular mechanism of how Avr2 compromises plant immunity requires identification of the Avr2 host target, which has so far proven challenging (Ma *et al*., 2015). Improvements in technology (e.g. sensitivity of mass spectrometry), and the results of this study suggesting that Avr2 likely targets an early component of PTI signaling at or near the plasma membrane, offers new opportunities to identify this target, and will be the subject of future work. Avr2 target identification, followed by molecular characterization of action, may offer new opportunities for the control of *Fusarium* disease in tomato.

## Author contributions

F.L.W.T. and M.J.B. designed the study; X.D., L.C., R.K.H. and N.T. performed the experiments; X.D., L.C., R.K.H., F.L.W.T. and M.J.B. analysed the data; and X.D., F.L.W.T. and M.J.B. together wrote the manuscript.

## Supporting information

Please note: Wiley Blackwell are not responsible for the content or functionality of any Supporting Information supplied by the authors. Any queries (other than missing material) should be directed to the *New Phytologist* Central Office.

**Fig. S1** *ΔspAvr2* ^*R45H*^ complements the virulence defect of a *FolΔAvr2* strain.
**Fig. S2** Side‐by‐side representations of the structures of Avr2 and its structural homologs: human Speckle‐type POZ protein, human TRAF6 and human SIAH1.
**Fig. S3** Overlays of the structure of Avr2 with structural homologs; Ptr‐ToxA, human Speckle‐type POZ protein, human TRAF6 and human SIAH1. Side view.
**Fig. S4** Overlays of the structure of Avr2 with structural homologs; Ptr‐ToxA, human Speckle‐type POZ protein, human TRAF6 and human SIAH1. Bottom view.
**Fig. S5** Subcellular localization of GFP‐tagged Avr2 and variants carrying targeting signals.
**Table S1** Primer sequences
**Methods S1** Infections assays of tomato and Arabidopsis plants and Avr2 mutant design.
