# Supplementary material for: Structure–function analysis of the Fusarium oxysporum Avr2 effector allows uncoupling of its immune‐suppressing activity from recognition
Source: New Phytol. 2017 Aug 31;216(3):897–914. doi: 10.1111/nph.14733 (PMC5659127; doi:10.1111/nph.14733)
Supplement: Supplementary file 1 — Fig. S1 ΔspAvr2 R45H complements the virulence defect of a FolΔAvr2 strain. Fig. S2 Side‐by‐side representations of the structures of Avr2 and its structural homologs: human Speckle‐type POZ protein, human TRAF6 and human SIAH1. Fig. S3 Overlays of the structure of Avr2 with structural homologs; Ptr‐ToxA, human Speckle‐type POZ protein, human TRAF6 and human SIAH1. Side view. Fig. S4 Overlays of the structure of Avr2 with structural homologs; Ptr‐ToxA, human Speckle‐type POZ protein, human TRAF6 and human SIAH1. Bottom view. Fig. S5 Subcellular localization of GFP‐tagged Avr2 and variants carrying targeting signals. Table S1 Primer sequences Methods S1 Infections assays of tomato and Arabidopsis plants and Avr2 mutant design. [file NPH-216-897-s001.pdf]

New Phytologist **Supporting Information**

Article title: **Structure-function analysis of the *Fusarium oxysporum* Avr2 effector allows uncoupling of its immune-suppressing activity from recognition**

Authors: Xiaotang Di, Lingxue Cao, Richard K Hughes, Nico Tintor, Mark J Banfield* and Frank LW Takken*

Article acceptance date: 03 July 2017

The following Supporting Information is available for this article:

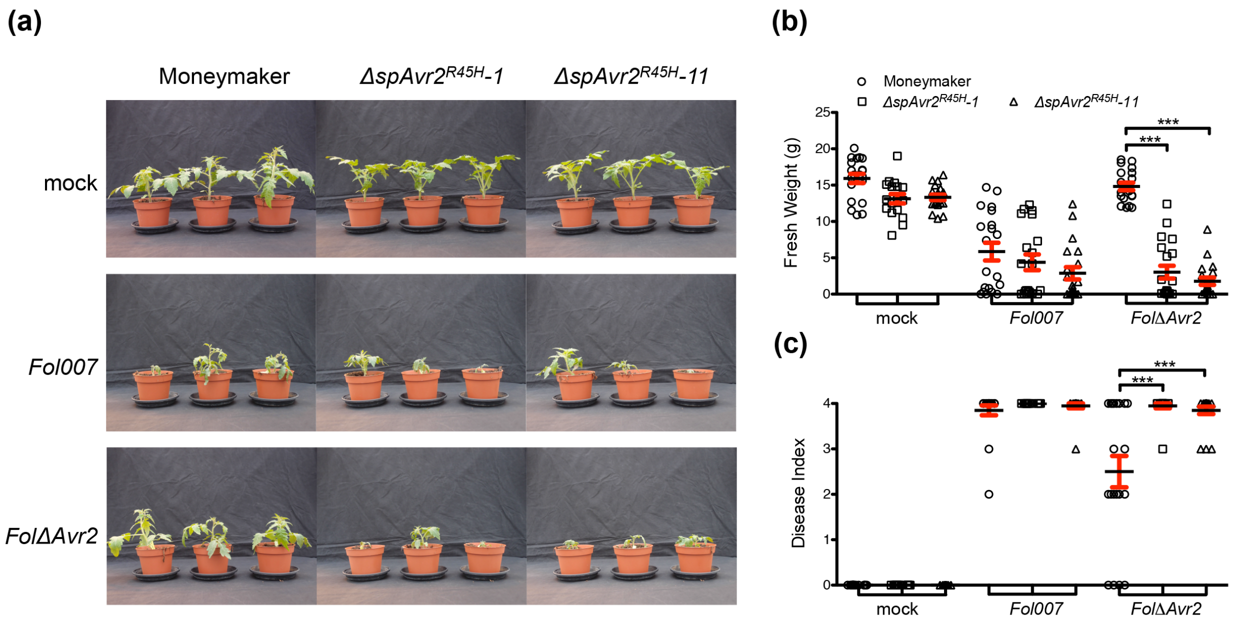

Fig. S1 *ΔspAvr2*^{R45H} complements the virulence defect of a *FolΔAvr2* strain.

ΔspAvr2^{R45H} complements the virulence defect of a *FolΔAvr2* strain (**a**) 10-day-old seedlings of wild type (Moneymaker), *ΔspAvr2*^{R45H}*-1* and *ΔspAvr2*^{R45H}*-11* transgenic tomato plants were inoculated with water (mock), wild-type Fusarium *Fol007* or *Fol ΔAvr2*. Three weeks after inoculation, (**b**) mean plant weight and (**c**) average disease index of 20 plants were scored. Error bar represent means ± SE (***P<0.001 one-way ANOVA). The experiments have been repeated twice with similar results.

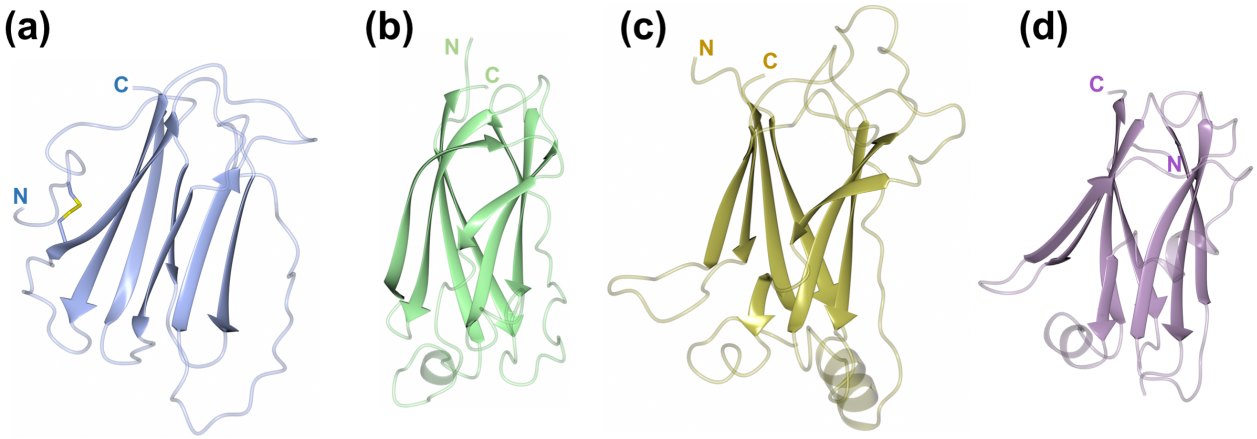

Fig. S2 Side-by-side representations of the structures of Avr2 and its structural homologs: human Speckle-type POZ protein, human TRAF6 and human SIAH1

Side-by-side representations of the structures of (a) Avr2, (b) human Speckle-type POZ protein, (c) human TRAF6 and (d) human SIAH1 (SINA domain of E3 ubiquitin ligase, Absentia Homolog 1) showing the similar overall protein folds. The orientation of Avr2 is as in Fig. 6a. The N- and C-termini are labelled, and non β-strand regions are transparent for clarity.

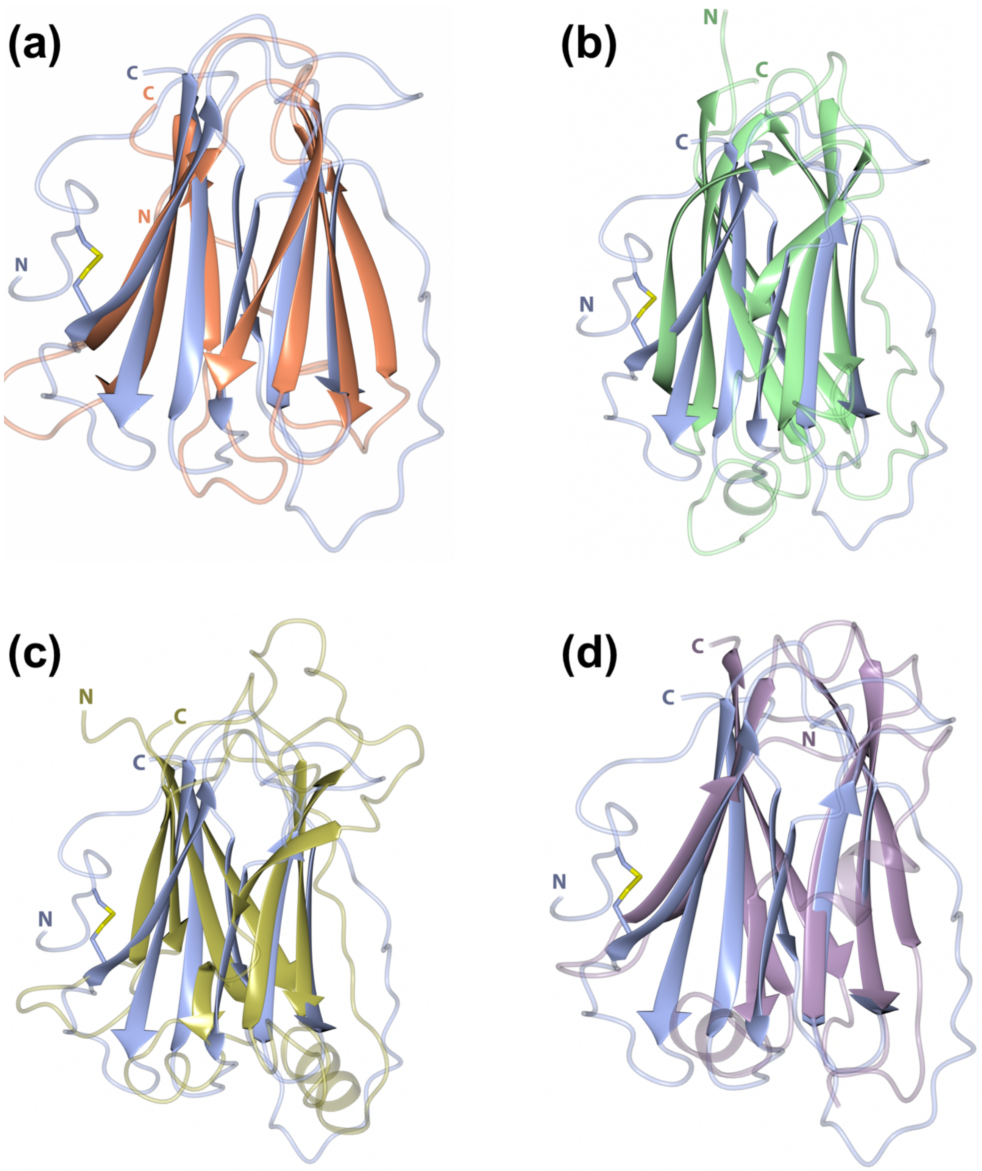

Fig. S3 Overlays of the structure of Avr2 with structural homologs; Ptr-ToxA, human Speckle-type POZ protein, human TRAF6 and human SIAH1. Side view.

Overlays of the structure of Avr2 with (a) Ptr-ToxA, (b) human Speckle-type POZ protein, (c) human TRAF6 and (d) human SIAH1 (SINA domain of E3 ubiquitin ligase, Absentia Homolog 1). The orientation of Avr2 is as in Fig. 6a. The N- and C-termini are labelled, and non β-strand regions are transparent for clarity.

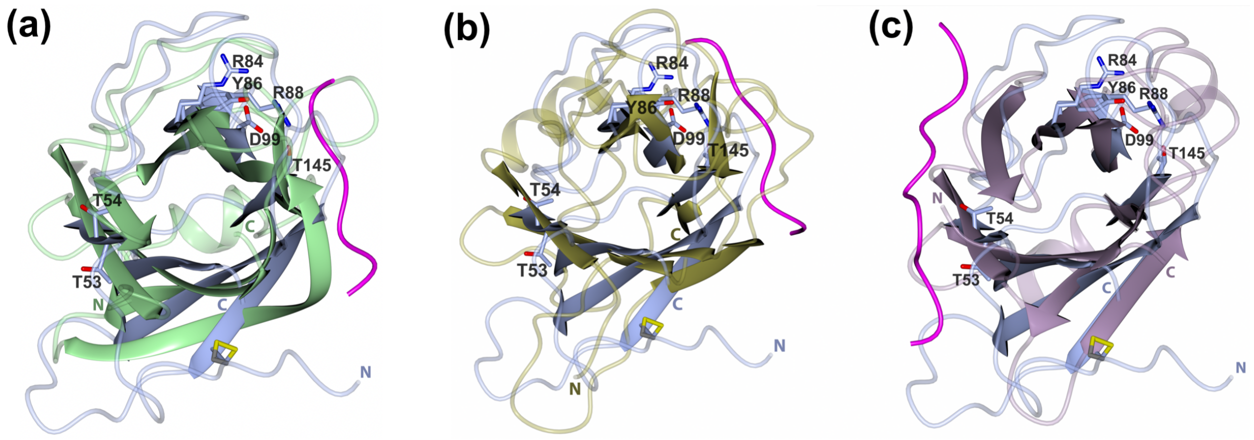

Fig. S4 Overlays of the structure of Avr2 with structural homologs; Ptr-ToxA, human Speckle-type POZ protein, human TRAF6 and human SIAH1. Bottom view. Overlays of the structure of Avr2 with (a) human Speckle-type POZ protein, (b) human TRAF6 or (c) human SIAH1 (SINA domain of E3 ubiquitin ligase, Absentia Homolog 1). The structures are viewed from the bottom of the barrels in suitable orientations, and with bound peptides in magenta. The location of the Avr2 mutations in relation to the bound peptides are shown. The N- and C-termini are labelled, and non β-strand regions are transparent for clarity.

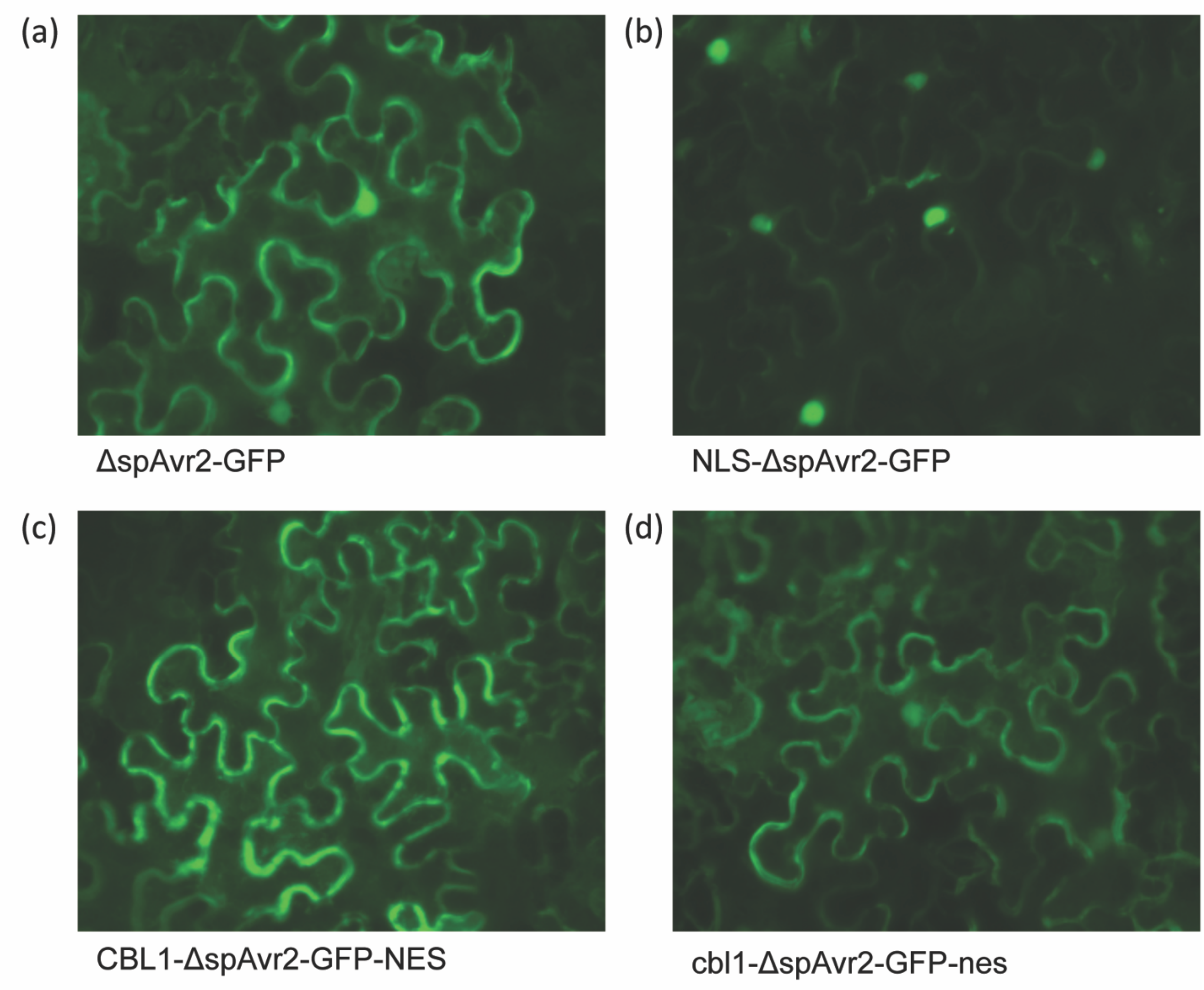

Fig. S5. Subcellular localization of GFP-tagged Avr2 and variants carrying targeting signals.

Subcellular localization of GFP-tagged Avr2 and variants carrying targeting signals. The following Avr2 variants were transiently expressed in *N. benthamiana*: (a) *ΔspAvr2-GFP*, (b) *NLS-ΔspAvr2-GFP*, (c) *CBL1-ΔspAvr2-GFP-NES* and (d) *cbl1-ΔspAvr2-GFP-nes* and their localization pattern was observed with a epifluorescence microscope. Nuclear localized GFP signals were observed in all samples except in (c). Leaf discs from this experiment were used to asses flg22-triggered ROS production (Figure 7A).. The N- and C-termini are labelled, and non β-strand regions are transparent for clarity.

Table S1 Primers used in this study

Primer name	Target gene	Sequences (5'-3')
FP873	attb	GGGGACCACTTTGTACAAGAAAGCTGGGT
FP872	attb	GGGGACAAGTTTGTACAAAAAAGCAGGCT
FP963	Avr2	CAATCCTCTGAGATAGTAAG
FP962	Avr2	TGAGCGGGCTGGCAATTC
FP2525	Avr2	CGCTCTAGAATGCCTGTGGAAGATGCCGAT
FP2848	Avr2	ACTGATTGTGGCTGGACCTC
FP2849	Avr2	GGACTGAATCACCGCATTTACGA
FP2274	Avr2	GCGGGATCCTCCATCCTCTGAGATAGTAAG
FP3147	AtActin	GAGCTGTGTTTCCTAGTATTGTGGG
FP3148	AtActin	CAAGATCAAGACGTAGGATAGCATG
FP6915	$Avr2^{T145E\text{-}F}$	GCTCTCGAGGTCCAGCCGAGATCAGTTGGGATGCCGA
FP6916	$Avr2^{T145E\text{-}R}$	TCGGCATCCCAACTGATCTCGGCTGGACCTCGAGAGC
FP6917	$Avr2^{T145K\text{-}F}$	GCATCCCAACTGATTTTGGCTGGACCTCGAG
FP6918	$Avr2^{T145K\text{-}R}$	CTCGAGGTCCAGCCAAAATCAGTTGGGATGC
FP6919	$Avr2^{D99A\text{-}F}$	GGGGGGCGACAGCAATGACAGTGCGGAG
FP6920	$Avr2^{D99A\text{-}R}$	CTCCGCACTGTCATTGCTGTCGCCCCCC
FP6921	$Avr2^{D99E\text{-}F}$	GGGGGGGCGACCTCAATGACAGTGCGGA
FP6922	$Avr2^{D99E\text{-}R}$	TCCGCACTGTCATTGAGGTCGCCCCCCC
FP6923	$Avr2^{T54R\text{-}F}$	GGTGCTGAAGCTCCTAGTAAATGAAGTAGAAGACGTGC
FP6924	$Avr2^{T54R\text{-}R}$	GCACGTCTTCTACTTCATTTACTAGGAGCTTCAGCACC
FP6925	$Avr2^{R88A\text{-}F}$	CTCCAACGCGACTTGCTTCGTAAATGCGGTGATTCAGTCC
FP6926	$Avr2^{R88A\text{-}R}$	GGACTGAATCACCGCATTTACGAAGCAAGTCGCGTTGGAG
FP6927	$Avr2^{Y86A\text{-}F}$	GCGACTTCGTTCGGCAATGCGGTGATTCAGTCCCGAAT
FP6928	$Avr2^{Y86A\text{-}R}$	ATTCGGGACTGAATCACCGCATTGCCGAACGAAGTCGC
FP6929	$Avr2^{T53R\text{-}F}$	TGCTGAAGCTCGTCCTAAATGAAGTAGAAGACGTGCGGCG

FP6930	Avr2^{T53R-R}	CGCCGCACGTCTTCTACTTCATTTAGGACGAGCTTCAGCA
FP6931	Avr2^{R84A-F}	GCGACTTCGTTCGTAAATGGCGTGATTCAGTCCCGAATTG
FP6932	Avr2^{R84A-R}	CAATTCGGGACTGAATCACGCCATTTACGAACGAAGTCGC

Methods S1 Infections assays of tomato and Arabidopsis plants and Avr2 mutant design.

V. dahliae inoculation assay tomato

10-day-old tomato plants were carefully uprooted from the soil and the roots were placed in a race 1 *V. dahliae* JR2 inoculum (10^6 conidia/ml) for 5 min (Fradin *et al.*, 2009). Thereafter, the plants were transferred to fresh soil. 14 days post inoculation plants were photographed and disease symptoms were scored. The canopy area of plants was measured with Image J software and a one-way ANOVA was performed with PRISM 5.0 statistics software (Fradin *et al.*, 2009).

B. cinerea inoculation assay tomato

B. cinerea strain B05.10 was grown on Malt Extract Agar (Oxoid, Basingstoke, UK; 50 g/l) in the dark at 20 °C for 3-4 days. The plates were placed for one night under near-UV light (350–400 nm) and returned to darkness to promote sporulation. Spores were harvested 4-7 days later in 20 mL of water and filtered over glass wool to remove mycelia. The conidia were pelleted (5 min, 120 g) and resuspended in Potato dextrose Broth at 5×10^6 conidia/ml. Two microliter droplets of *B. cinerea* spore suspension were inoculated on dissected leaves of six-week-old tomato plants (Zhang & Van Kan, 2013). Pictures were captured at 3 dpi and lesion diameters were quantified by Image J.

Pst DC3000 inoculation assay tomato an Arabidopsis

Pst DC3000 was grown at 28°C for 48 h on King's B liquid medium (KB) containing 40 µg/ml rifampicin (King *et al.*, 1954). Bacteria were collected by centrifugation (5 min, 1000 g) and resuspended in 1 ml of 10 mM $MgSO_4$ to an OD600 of 0.0005 and syringe-infiltrated into leaves of either four-week-old tomato or *A. thaliana* plants. Arabidopsis leaf discs (5 mm diameter) were harvested at 0, 1, 2 dpi and tomato discs were collected at 3 dpi. Bacteria were extracted in

10 mM $MgSO_4$ and serial dilutions were plated on King's B medium containing 40 µg/ml rifampicin (Dong *et al.*, 1991). Viable colonies were counted after 2 day incubation at 28°C.

***F. oxysporum* and *V. dahliae* bioassays in Arabidopsis**

For *F. oxysporum* bioassays 14-day-old Arabidopsis seedlings were uprooted and the roots were cleaned with tap water. The seedlings were placed for 5 min in a *Fo5176* spore suspension (10^6 spores/ml) generated from a five-day-old *Fo5176* liquid culture (100 mM KNO3, 3% sucrose and 0.17% YNB without amino acids). Inoculated seedlings were repotted and placed in a growth chamber with a 13/11dark/light regime at 28 °C. Disease index was scored for 20 plants/treatment, on a scale of 0 to 5 (Gawehns *et al.*, 2014).

V. dahliae JR2 assays spores were harvested from a five-day-old liquid culture (100 mM KNO3, 3% sucrose and 0.17% YNB without amino acids) by filtering over three layers of micro-cloth. The seedlings were placed in the *V. dahliae* spore suspension (10^6 spores/ml) for 5 min and replanted. Disease symptoms developed after two- to three weeks and the disease index was scored at 21 dpi.

Design of Avr2 mutants for functional assays

To design mutations in Avr2, we first searched to identify putative functionally relevant sites on the protein. For this, we selected a subset of structures listed in the DaliLite output that showed structural homology to Avr2, and which had ligands bound (these were exclusively peptide ligands). Three representative structures that had peptides bound at one of two sites, Speckle-type POZ (SPOP) protein and TRAF6 (peptide at the same site) and SIAH1 (peptide bound at a second site) were selected for further analysis. The structures of these proteins were overlaid on Avr2 (by DaliLite), and compared to suggest residues for mutation in Avr2 assuming, in the absence of any other knowledge, that similar ligand interaction sites may be important for Avr2 function. To perturb the SIAH1-based interaction site we designed mutations $Avr2^{T53R}$ and $Avr2^{T54R}$ to deliver steric constraints to ligand binding. To perturb the Speckle-type POZ (SPOP) protein/TRAF6 interaction site we designed mutations $Avr2^{R84A}$, $Avr2^{Y86A}$, $Avr2^{R88A}$, $Avr2^{D99A}$, $Avr2^{D99E}$, $Avr2^{T145E}$, $Avr2^{T145R}$, to either remove side-chains that could mediate potential ionic or hydrogen bonding interactions with ligands at this site (alanine mutants), or deliver steric constraints to ligand binding.

references

Dong X, Mindrinos M, Davis KR, Ausubel FM. 1991. Induction of Arabidopsis defense genes by virulent and avirulent Pseudomonas syringae strains and by a cloned avirulence gene. *Plant Cell* **3**(1): 61-72.

Fradin EF, Zhang Z, Juarez Ayala JC, Castroverde CD, Nazar RN, Robb J, Liu CM, Thomma BP. 2009. Genetic dissection of Verticillium wilt resistance mediated by tomato Ve1. *Plant Physiol* **150**(1): 320-332.

Gawehns F, Houterman PM, Ichou FA, Michielse CB, Hijdra M, Cornelissen BJC, Rep M, Takken FLW. 2014. The *Fusarium oxysporum* effector Six6 contributes to virulence and suppresses I-2-mediated cell death. *Mol Plant Microbe In* **27**(4): 336-348.

King EO, Ward MK, Raney DE. 1954. Two simple media for the demonstration of pyocyanin and fluorescin. *Journal of Laboratory and Clinical Medicine* **44**(2): 301-307.

Zhang LS, Van Kan JAL. 2013. *Botrytis cinerea* mutants deficient in D-galacturonic acid catabolism have a perturbed virulence on *Nicotiana benthamiana* and Arabidopsis, but not on tomato. *Mol Plant Pathol* **14**(1): 19-29.
